# Combating Concomitant Bacterial and Fungal Infections via Codelivery of Nitric Oxide and Fluconazole

**DOI:** 10.1021/acsami.5c00174

**Published:** 2025-04-14

**Authors:** Rashmi Pandey, Natalie Crutchfield, Mark Richard Stephen Garren, Ekaa Manohar Kasetty, Manjyot Kaur Chug, Elizabeth J. Brisbois, Hitesh Handa

**Affiliations:** † School of Chemical, Materials, and Biomedical Engineering, College of Engineering, University of Georgia, Athens, Georgia 30602, United States; ‡ Franklin College of Arts and Sciences, University of Georgia, Athens, Georgia 30602, United States; § Pharmaceutical and Biomedical Science Department, College of Pharmacy, University of Georgia, Athens, Georgia 30602, United States

**Keywords:** nitric oxide, fluconazole, drug delivery, antibacterial, antifungal, hospital-acquired infections

## Abstract

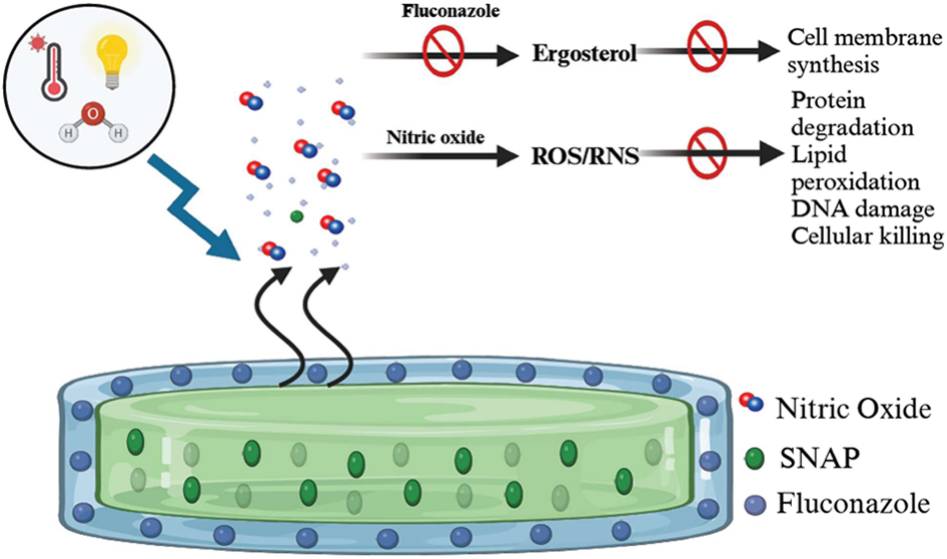

Device-associated infections are a major challenge for healthcare and cause patient morbidity and mortality as well as pose a significant economic burden. Infection-causing bacteria and fungi are equally notorious and responsible for biofilm formation and the development of antibiotic and antifungal-resistant strains. Biomaterials resisting bacterial and fungal adhesion can address device-associated infections more safely and efficiently than conventional systemic antibiotic therapies. Herein, we present a combination of potent antibacterial nitric oxide (NO) with antifungal fluconazole codelivery system from a polymeric matrix to combat bacterial and fungal infections simultaneously. The NO donor *S*-nitroso-*N*-acetyl-penicillamine (SNAP)-blended low-water-uptake polycarbonate urethane (TSPCU) was dip-coated with high-water-uptake polyether urethane (TPU) containing fluconazole to have an antibacterial and antifungal surface. The composites were characterized for surface wettability and coating stability using water contact angle (WCA) analysis. The real-time NO release (72 h) was evaluated using a chemiluminescence-based nitric oxide analyzer which showed physiologically relevant levels of NO released. The composites released fluconazole for 72 h under physiological conditions. Antibacterial analysis demonstrated a > 3-log reduction of viable *Staphylococcus aureus* and >2-log reduction of viable *Escherichia coli* compared to controls. The antifungal evaluation resulted in ∼98% reduction in adhered and ∼92% reduction in planktonic *Candida albicans*. The SNAP-fluconazole composites also showed biocompatibility against mouse fibroblast cells. This novel preventative strategy to combat bacterial and fungal infections may offer a promising tool for further translational research.

## 1 Introduction

Invasive medical devices are an integral part of modern medicine and are crucial in saving lives and improving patient outcomes. Infection of medical devices by microorganisms is a major contributor to hospital-acquired infections (ca. 50–70%) and presents a critical challenge for patient care.^1^ Microorganisms can colonize a device surface within a few hours of application and eventually form a closed community of microcolonies embedded inside an extracellular polymeric matrix, known as a biofilm. Biofilms can evade antibiotic therapy and the host immune system making them extremely difficult to eradicate. Systemic antibiotic treatment is given to treat infections in clinical settings.^2^ Biofilms prevent the diffusion of antibiotics and can require up to 10–1000 times higher concentrations for treatment of the infection. This phenomenon also has a vital role in the widespread emergence of antibiotic-resistant microorganisms.^2−4^

The fungal strain *Candida albicans* is the fourth leading cause of vascular catheter-related infections and the third leading cause of urinary catheter-related infections and has been shown to form matured biofilm in less than 24 h.^5^ Additionally, clinically relevant infections are rarely caused by a single microbial type. Most infections are polymicrobial and require treatment for both bacterial and fungal species. Unfortunately, fungal infections are usually overlooked in clinical settings where antibiotic therapy is the gold standard for any infection presented.^6^ Fluconazole, a first-generation, orally administered, water-soluble, nontoxic triazole derivative, is easily absorbed and is an effective antifungal agent.^7^ The family of azole antifungal agents act by inhibiting the fungal cytochrome P_450_ enzyme 14 α-demethylase, stopping the conversion of lanosterol to ergosterol. Ergosterol is the main sterol of yeast and fungal cell membranes, and its absence causes membrane degradation.^8^ Fluconazole has been used for battling infections in peritoneal dialysis, where it showed higher efficiency than other antifungal agents.^9^ Fluconazole has also been successfully used as an antifungal treatment against *Candida* infections in critically ill patients undergoing surgery,^10^ and as a prophylactic treatment in very low birth weight infants with central vascular access,^11^ critically ill surgical patients,^12^ and immunosuppressed bone marrow transplant patients.^13^ Moreover, fluconazole has shown synergistic fungal killing when combined with other antibiotics, making it highly clinically relevant.^14^ Recent reports with the incorporation of azole-based antifungals into high-water-uptake nanofibers have shown promising antifungal effects both *in vitro* and *in vivo*.^15,16^

The gasotransmitter nitric oxide (NO), an endogenously produced free radical, has been recognized as a broad-spectrum antibacterial and antithrombotic agent. NO is involved in multiple physiological processes, including vasodilation, hemostasis, endothelium functionality, and promotion of wound healing.^17,18^ NO can produce highly reactive oxygen and nitrogen species (ROS and RNS) in the presence of oxygen. These ROS and RNS species including hydroxyl radical (OH^–^), peroxynitrite (ONOO^–^), nitrogen dioxide (NO_2_), and dinitrogen tetroxide (N_2_O_3_) can exert bactericidal effects via damaging cellular proteins, DNA, and lipids. They can introduce DNA cleavage, lipid peroxidation, membrane, and protein damage, eventually killing the bacterial cell.^19,20^ The multimechanistic mode of NO antibacterial action prevents bacteria from developing resistance,^20−22^ making it an attractive alternative to antibiotics. The bioactivity of NO is highly dose-dependent, making it critical to control the NO payload that is being delivered.^23^ Various NO-releasing biomaterials have been developed via encapsulation,^23^ physical blending,^24^ solvent swelling,^25^ and covalent immobilization^26^ of NO donors with polymeric platforms. However, NO-releasing materials only exhibit modest activity against fungal infections.^27^ The NO donor *S*-nitroso-*N*-acetyl-penicillamine (SNAP) has been shown to have negligible effects against *C. albicans* when tested at 5 mM concentration^28^ which is 10 times higher than the cytocompatible levels for mammalian cells (∼0.5 mM).^25^

Interestingly, NO has shown synergistic antifungal capabilities with azole-based antifungal drugs like fluconazole.^5,29^ However, a combinatorial polymeric surface with codelivery of these drugs and NO has not been reported for biomedical applications. Wang et al. have recently reported the synthesis of NO-releasing azole drugs with varying degrees of *in vitro* and *in vivo* effects on cryptococcal meningitis, with some structures outperforming fluconazole.^30^ They also demonstrated that NO-releasing drugs had enhanced effects on matured biofilms compared to fluconazole. However, the designed compounds were species-specific and did not significantly affect *C. albicans* growth. Additionally, Madariaga-Venegas et al, showed that NO-releasing aspirin could enhance fluconazole activity against otherwise resistant *Candida* species strains.^31^ Recently, Li et al. have demonstrated a synergistic eradication of *C. albicansvia* codelivery of NO and clotrimazole using α-cyclodextrin-modified polydopamine nanoparticles, supporting the viability of this study.^32^ The major mechanism of eradicating fungal infection is phagocytosis via macrophages.^28^ NO produced by activated macrophages are known to enhance phagocytosis capabilities against fungal strains.^33^ Peroxynitrite produced by macrophages is also shown to be responsible for fungal killing.^34^ Furthermore, NO-releasing aspirin could prevent hyphae formation in *C. albicans*, a critical virulence factor for the fungus.^31^ Inhibition of fungal hyphae formation could be a potential mechanism for NO antifungal activity.

This study presents a novel strategy for designing a cytocompatible biomaterial with the potential for codelivery of antibacterial NO and antifungal fluconazole. The NO donor SNAP was blended into a low-water-uptake thermoplastic silicone polycarbonate urethane (TSPCU) and top-coated with high-water-uptake thermoplastic polyurethane (TPU) blended fluconazole to fabricate composites that could release NO and fluconazole simultaneously. These composites were evaluated for static water contact angle changes, water uptake, and stability. They were further characterized for NO and fluconazole release under physiological conditions. The fabricated composites were evaluated for cytocompatibility with mammalian cells followed by antibacterial and antifungal properties against *Escherichia coli*, *Staphylococcus aureus*, and*C. albicans* in 24 h studies.

## 2 Materials and Methods

### 2.1 Materials

All of the reagents were used in the commercially available forms unless otherwise mentioned. *N*-Acetyl-d-penicillamine (NAP), ethylenediaminetetraacetic acid (EDTA), and tetrahydrofuran (THF) were purchased from Sigma-Aldrich (St. Louis, MO). Fluconazole (>98% purity) was procured from TCI America (Portland, OR). Methanol, hydrochloric acid, and sulfuric acid were obtained from Fisher Scientific (Pittsburgh, PA). The polymeric resin thermoplastic silicone polycarbonate polyurethane ChronoSil AL 75A (10%) (TSPCU) was procured from AdvanSource Biomaterials (Wilmington, MA), and thermoplastic polyurethane Tecoflex SG-80A (TPU) was from Lubrizol Advanced Materials, Inc. (Cleveland, OH). The mouse fibroblast cell line (NIH-3T3, CRL-1658), *S. aureus* (ATCC 6538), *E. coli* (ATCC 25922), and *C. albicans* (ATCC MYA4441) were revived from stocks originally obtained from American Type Cell Culture (ATCC). Yeast Media was from Fisher Scientific. Dulbecco’s modified Eagle medium (DMEM) was purchased from Gibco. 3-[4,5-Dimethylthiazol-2-yl]-2,5 diphenyl tetrazolium bromide (MTT), dimethyl sulfoxide (DMSO), penicillin–streptomycin, trypsin-EDTA, phosphate buffer saline (PBS), tryptic soy (TS), and Luria–Bertani (LB) broth, and agar were purchased from Sigma-Aldrich (St. Louis, MO). Fetal bovine serum was from VWR Life Science. All aqueous solutions were prepared in Milli-Q water (18.2 MΩ) and purified using a Mettler Toledo (Columbus, OH) apparatus. PBS buffer (10 mM, pH 7.4) was prepared by dissolving sodium chloride (139 mM), potassium chloride (2.68 mM), sodium phosphate monobasic (1.8 mM), and sodium phosphate dibasic (8.2 mM) in 1 L of Milli-Q water. PBS was supplemented with 100 μM of EDTA to chelate out metal ions and labeled as PBS-EDTA. The buffer was autoclaved and stored under sterile conditions for all of the biological experiments.

### 2.2 Material Fabrication

#### 2.2.1 SNAP Synthesis

SNAP was synthesized following a method reported by Chipinda & Simoyi with some modifications.^35^ 5 g of *N*-acetylpenicillamine (NAP) was dissolved in 60 mL of methanol followed by a dropwise sequential addition of 20 mL of 12 N HCl and 5 mL of concentrated H_2_SO_4_. The solution was allowed to cool while stirring slowly. Separately, 5 g of NaNO_2_ was dissolved in 40 mL of DI water. Both solutions were mixed and cooled in an ice bath for 6–8 h to precipitate the SNAP crystals. The crystals were collected by vacuum filtration, washed with water, and allowed to air-dry. The SNAP crystals’ purity was measured using a nitric oxide analyzer (NOA) as described in Section 2.6. Only batches with >95% purity (i.e., moles of NO per mole of SNAP × 100%) were used. The reaction and crystals were protected from light and stored at −20 °C.

#### 2.2.2 Fabrication of NO-Releasing Polymeric Composites

A thermoplastic silicone polycarbonate urethane (TSPCU) resin (ChronoSil AL 75A (10%)) containing 10% silicone was used to fabricate the base hydrophobic composite. The resins were dried for 4 h at 80 °C to remove any moisture and dissolved in anhydrous THF at a concentration of 50 mg mL^–1^ via vigorous stirring at room temperature. SNAP was blended into the TSPCU solution at a concentration of 10 wt % of the total mass. The loading of SNAP was selected based on previous work done on low-water-uptake polymeric NO-releasing materials.^35−37^ The resulting polymeric solution was cast into Teflon molds and kept in a fume hood overnight to remove the THF. 15 mL of polymeric solution was cast on a Teflon mold of dimension 7 × 7 cm. The resulting composites had a thickness of ∼0.3 mm. A thermoplastic polyether urethane (TPU, Tecoflex SG-80A) with high water uptake was used for top coating the SNAP-incorporated TSPCU base. The resins were dried and dissolved in THF similar to the TSPCU. Varying concentrations of fluconazole were blended into the TPU solution (1, 5, 10, 20, and 40 wt % of total polymeric mass). The SNAP-blended TSPCU composites were cut into small circular disks with 8 mm diameter and dip-coated with fluconazole blended TPU. The dip-coats were applied thrice with 1 h of drying in between the coats. The polymeric composites were further dried in a fume hood to allow the evaporation of the solvent. The composites were further desiccated for ∼18 h to remove residual solvent. The resulting composites with a thickness of ∼0.35 mm were stored at −20 °C with desiccant and protected from light. The samples fabricated for this study are listed in Table 1.

**Table 1:** Sample Fabrication Details

sample	fabrication details
TSPCU control	50 mg mL^–1^ TSPCU + 50 mg mL^–1^ TPU top coat (3×)
SNAP	50 mg mL^–1^ TSPCU with SNAP (10 wt %) + 50 mg mL^–1^ TPU top coat (3×)
Fluconazole	50 mg mL^–1^ TSPCU + 50 mg mL^–1^ TPU with fluconazole (1, 5, 10, 20, or 40 wt %) top coat (3×)
SNAP-Fluconazole	50 mg mL^–1^ TSPCU with SNAP (10 wt %) + 50 mg mL^–1^ TPU with fluconazole (1, 5, 10, 20, or 40 wt %) top coat (3×)

### 2.3 Physical Characterizations

#### 2.3.1 Surface Wettability Evaluation

The effects of SNAP blending and TPU coating on the TSPCU base composites were evaluated using an Ossila Contact Angle Goniometer (Model: L2004A1). TPU is relatively hydrophilic compared to TSPCU and may induce some changes to surface wettability. Moreover, SNAP incorporation is also known to influence the surface wettability of hydrophobic polyurethanes. A droplet of DI water (10 μL) was placed on the composite surface, and the static water contact angle was recorded.

#### 2.3.2 Top-Coat Stability

Static water contact angles of the composites were measured to assess the stability of the hydrophilic TPU top coat on hydrophobic TSPCU. The composites were soaked in PBS and incubated at 37 °C under shaking conditions for 24 h to mimic the *in vitro* biological assays. After incubation, the composites were removed from PBS and gently dabbed with tissue paper to remove excess PBS, and the static water contact angle was measured as described in Section 2.3.1.

#### 2.3.3 Water Uptake

Water uptake of the polymeric composites was evaluated for 24 h. The composites were weighed and soaked in DI water and incubated at 37 °C in a Thermo Scientific incubator. The next day the composites were dabbed with a KimWipe and the masses were recorded again. Water uptake was calculated using eq 1:
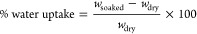
1where *w*_soaked_ is the weight of soaked composites and *w*_dry_ is the dry weight.

#### 2.3.4 X-ray Diffraction (XRD)

Crystalline properties of fluconazole blended TPU were evaluated using X-ray diffraction spectroscopy. Thin composites were fabricated for fluconazole blended TPU (1, 5, 10, 20, and 40 wt %) without the TSPCU base layer and the diffraction peaks were measured using a Bruker D8 Advance X-ray diffractometer Cu–Kα radiation (λ = 1.54 Å). A Si P-type B-doped zero diffraction plate was used to level the samples in the holder.

#### 2.3.5 Scanning Electron Microscopy (SEM)

The surface morphology of the fabricated composites was studied using field emission scanning electron microscopy (SEM, FEI Teneo, FEI Co.). The samples were coated with 10 nm of gold–palladium using a Leica sputter coater. The imaging was performed at an accelerating voltage of 10.0 kV and a working distance of 10.0 mm.

#### 2.3.6 Attenuated Total Reflectance-Fourier Transform Infrared (ATR-FTIR) Spectroscopy

ATR-FTIR spectroscopy was performed on SNAP and fluconazole-integrated samples to confirm integration of the compounds for controlled NO and fluconazole release using a Spectrum 3 Spectrometer (PerkinElmer). Composites as well as base materials including polyurethanes, SNAP, and fluconazole were studied. Spectra were collected across 4000–650 cm^–1^ at a resolution of 4 cm^–1^ averaged over 64 scans. Spectral analysis was performed using Spectrum 10 software (PerkinElmer).

#### 2.3.7 Raman Spectroscopy

The structure of the fluconazole was analyzed using Raman spectroscopy to determine the polymorphic form. The Raman spectroscopy was completed using a Thermo Scientific DXR Raman microscope using 8 mW power at 532 nm from 2900 to 3200 cm^–1^.

### 2.4 Fluconazole Release

Fluconazole release from the samples under physiological conditions was examined using UV–vis spectroscopy. Briefly, material composites (surface area ∼ 1 cm^2^) were immersed in 1 mL of PBS supplemented with EDTA (100 μM) and incubated at 37 °C in a Thermo Scientific incubator, protected from light. At each time point, the leachate was removed, transferred to a quartz cuvette, and the absorbance was read at 203 nm using an Agilent UV–vis spectrophotometer. After each measurement, fresh buffer was added to the sample container to keep the total buffer volume unchanged throughout the experiment. Fluconazole concentration in the buffer was determined from the absorbance value for each sample and a predetermined calibration curve for known concentrations of fluconazole in PBS-EDTA buffer. The calibration curve is shown in Figure S4.

Calculations of cumulative fraction of release were performed based on the total molar amount of fluconazole released into buffer at various time points (*M*_*t*_) with respect to the total mass of fluconazole loaded (*M*_0_) (eq 2). Based on fractions of *M*_*t*_/*M*_0_< 0.6, a diffusion-controlled model assuming the validity of Fick’s second law of diffusion was fitted to estimate diffusion coefficients (*D*) for the different fluconazole formulations based on prior work with slab-like devices:^38^
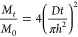
2

### 2.5 SNAP Diffusion

Total SNAP leaching from the composites under physiological conditions was examined using a Thermo Scientific Genesis 10 S UV–visible spectrophotometer. The circular composites (*n* = 5) were immersed in 1 mL of PBS (with 100 μM EDTA) and incubated at 37 °C, protected from light. At different time intervals, the absorbance of the solution was determined using a UV–vis spectrophotometer. SNAP has a distinct peak at 340 nm which was used to quantify the amount of SNAP diffused into the solution.^39^ After each measurement, fresh buffer was added to the sample container to keep the total buffer volume unchanged throughout the experiment. SNAP concentration in the buffer was determined from the absorbance value for each sample and a predetermined calibration curve for known concentrations of SNAP in PBS-EDTA buffer. The calibration curve is shown in Figure S6.

### 2.6 Nitric Oxide Release

Real-time NO release from the SNAP-containing polymeric composites was measured using a Sievers Zysene Chemiluminescence Nitric Oxide Analyzer 280i (NOA) (Boulder, CO). The small circular composites with a surface area of ∼1 cm^2^ were submerged in an amber-colored chamber containing 3 mL of PBS (pH: 7.4) supplemented with 100 μM of EDTA. EDTA was added to chelate any interfering metal ions present in the solution. The sample chamber was placed in a water bath maintained at 37 °C to mimic physiological conditions. A sweep gas (nitrogen) was purged continuously at a rate of 200 mL min^–1^. The released NO is carried into the chemiluminescence detection chamber where it reacts with ozone and emits a photon, detected by the NOA.^40^ The reaction resulting in the conversion is as follows:
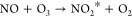

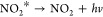
The NOA gives the concentration of NO in ppb which is converted into a standard NO release (×10^–10^ mol-NO cm^–2^ min^–1^) using the NOA calibration constant (mol-NO ppb^–1^ s^–1^) and surface area of the sample. NOA calibration constant was calculated using acidified sodium nitrite as standard. At least 5 replicates of each sample type were tested.

### 2.7 Antifungal Efficacy

To evaluate the antifungal capability of the fabricated composites, a 24 h fungal adhesion assay was performed as described elsewhere.^27,41^*C. albicans* was chosen as the model organism to evaluate the antifungal properties of the fabricated films. Yeast media (broth and agar) were prepared according to the manufacturer’s instructions by dissolving the powder in Milli-Q water and autoclaving for 30 min. Agar-containing media was poured into sterile Petri plates and allowed to solidify under sterile conditions. The broth was used for culturing the fungal cells, whereas the agar plates were used for CFU counting. The fungus was inoculated in yeast media and incubated at 37 °C in a shaker incubator shaking at 150 rpm. Cells in the exponential growth phase were washed in sterile PBS by centrifuging at 4400 rpm for 10 min. The resulting pellet was resuspended in sterile PBS and diluted to get a suspension with 1 × 10^8^ CFU mL^–1^ (corresponding to an absorbance of 0.1 at 600 nm). The polymeric composites were UV sterilized for 20 min on each side and exposed to the bacterial suspension (*n* = 3). The plates were sealed and incubated in a shaking incubator under physiological conditions, 37 °C, PBS, pH 7.4, and protected from light. After 24 h of incubation, the samples were removed and gently rinsed with PBS to remove any loosely attached fungal cells. Next, the adhered fungi were detached into fresh PBS by homogenizing the composites for 1 min at 30,000 rpm followed by vortexing for 30 s. The collected fungal suspension was further diluted in sterile PBS and plated on appropriate media containing agar. The plates were incubated overnight at 37 °C, and individual colonies obtained were counted. The total CFU obtained was calculated using eq 3:
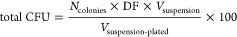
3where *N*_colonies_ is the number of colonies, DF is the dilution factor, *V*_suspension_ is the volume of the suspension (mL), and *V*_suspension-plated_ is the volume of suspension plated (mL).

The total CFUs were normalized with surface areas of the composites and the reduction in bacterial viability was calculated using the CFU cm^–2^ using eq 4:
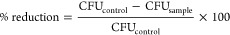
4

### 2.8 Antibacterial Efficacy

A Gram-positive bacteria *S. aureus*, and Gram-negative *E. coli* were chosen for this experiment. Bacterial media (LB for *E. coli* and TS for *S. aureus*) were prepared as described above. The bacterial colonies were inoculated in appropriate media and incubated at 37 °C in a shaker incubator shaking at 150 rpm to get to the exponential phase. The bacterial suspension was prepared as described in Section 2.7 and exposed to UV sterilized samples for 24 h under physiological conditions. The attached bacterial cells were detached and enumerated using colony counting as described above.

### 2.9 Cytotoxicity

Effects of the fabricated polymeric composites on mammalian cell viability were assessed on mouse fibroblast cells (NIH/3T3, ATCC-CRL-1658) using a cell cytotoxicity assay kit (CCK-8) based on a tetrazolium salt WST-8, which produces a water-soluble formazan dye upon reduction by cellular dehydrogenases. The intensity of the product is proportional to the number of viable cells. The protocol followed was in accordance with the International Standards Organization (ISO).^42^ Briefly, mouse 3T3 fibroblast cells were cultured in DMEM media supplemented with 10% FBS and 1% Penicillin–Streptomycin. Confluent cells (>80%) were detached from the culture flask using 0.25% trypsin-EDTA solution and seeded in a 96-well flat bottom plate at a density of 5 × 10^3^ cells per well. The plate was incubated at 37 °C with 5% CO_2_ overnight. The next day, the media was replaced with 100 μL of leachates from the respective polymeric composites (*n* = 5). The samples were sterilized before leachate preparation by UV irradiation for 30 min. Leachates were prepared by soaking one circular polymeric composite in 1 mL of complete DMEM at 37 °C and 5% CO_2_ overnight (surface area to volume ratio: 1 cm^2^ mL^–1^). The plates were further incubated for 24 h at 37 °C and 5% CO_2_. Cells without leachates were kept as positive control, only media was used as blank. For terminating the experiments, 10 μL of CCK reagent was added to the cells and incubated for 1 h at 37 °C and 5% CO_2_. Absorbance was measured at 450 nm. Cell viability was calculated using eq 5 after subtracting blank absorbance:
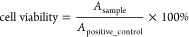
5where *A*_sample_ and *A*_positive_control_ are the absorbances of the sample and positive control, respectively.

### 2.10 Statistical Analysis

All of the data presented are mean ± standard deviation unless otherwise mentioned. All statistical analyses were performed using GraphPad Prism software (Version: 10.1.0). The samples were compared to TSPCU controls using one-way analysis of variance (ANOVA) followed by Tukey’s test. Results with *p* < 0.05 were deemed statistically significant.

## 3 Results and Discussion

### 3.1 Polymeric Composite Fabrication and Characterization

This study presents a novel biomaterial with dual antibacterial and antifungal capabilities achieved by combining NO and fluconazole release from the same interface. NO donor SNAP-blended low-water-uptake TSPCU composites were fabricated using solvent casting. SNAP was blended into TSPCU dissolved in THF at a concentration of 10 wt % of the polymer mass. The TSPCU ChronoSil AL 75A (10%) is a thermoplastic silicone polycarbonate polyurethane with <1% water uptake. NO donor SNAP has been incorporated extensively into such low-water-uptake polyurethanes and has shown great solubility, stability, and retention in the polymeric matrix. Wo et al.,^36^ demonstrated incorporation of >4 wt % of SNAP into a thermoplastic silicone-polycarbonate-urethane (CarboSil) matrix leads to crystal formation, enhancing NO release’s longevity. Higher concentrations of SNAP lead to the presence of both crystalline and amorphous SNAP which are responsible for the long-term and short-term release of NO, respectively. High concentrations of SNAP stabilize itself in the polymeric matrix via hydrogen bonding and slowly release NO over a longer period. Moreover, the hydrophobic nature of TSPCU could prevent rapid uptake of water into the polymeric matrix preventing rapid release and exhaustion of NO. 10 wt % of SNAP has been reported to have a sustained long-term release of NO under physiological conditions^25,37^ and was chosen for this study. The fabricated SNAP-incorporated composites were further coated with high-water-uptake thermoplastic polyurethane Tecoflex SG-80A. Fluconazole was blended into the bulk TPU at different concentrations *viz* 1, 5, 10, 20, and 40 wt %. The high-water uptake of TPU top coat allowed rapid penetration of water into the polymeric matrix and release of fluconazole. The antifungal drug must be released into the physiological environment to exert its bioactivity against fungal pathogens. The high water uptake of the TPU facilitates the release of fluconazole and acts as a barrier between the biomaterial and the surrounding microenvironment. The masses and thickness of the composites were recorded before and after dip-coats. The resulting composites were visibly smooth with a uniform thickness, as measured using a micrometer (∼0.35 mm).

**Figure 1:**
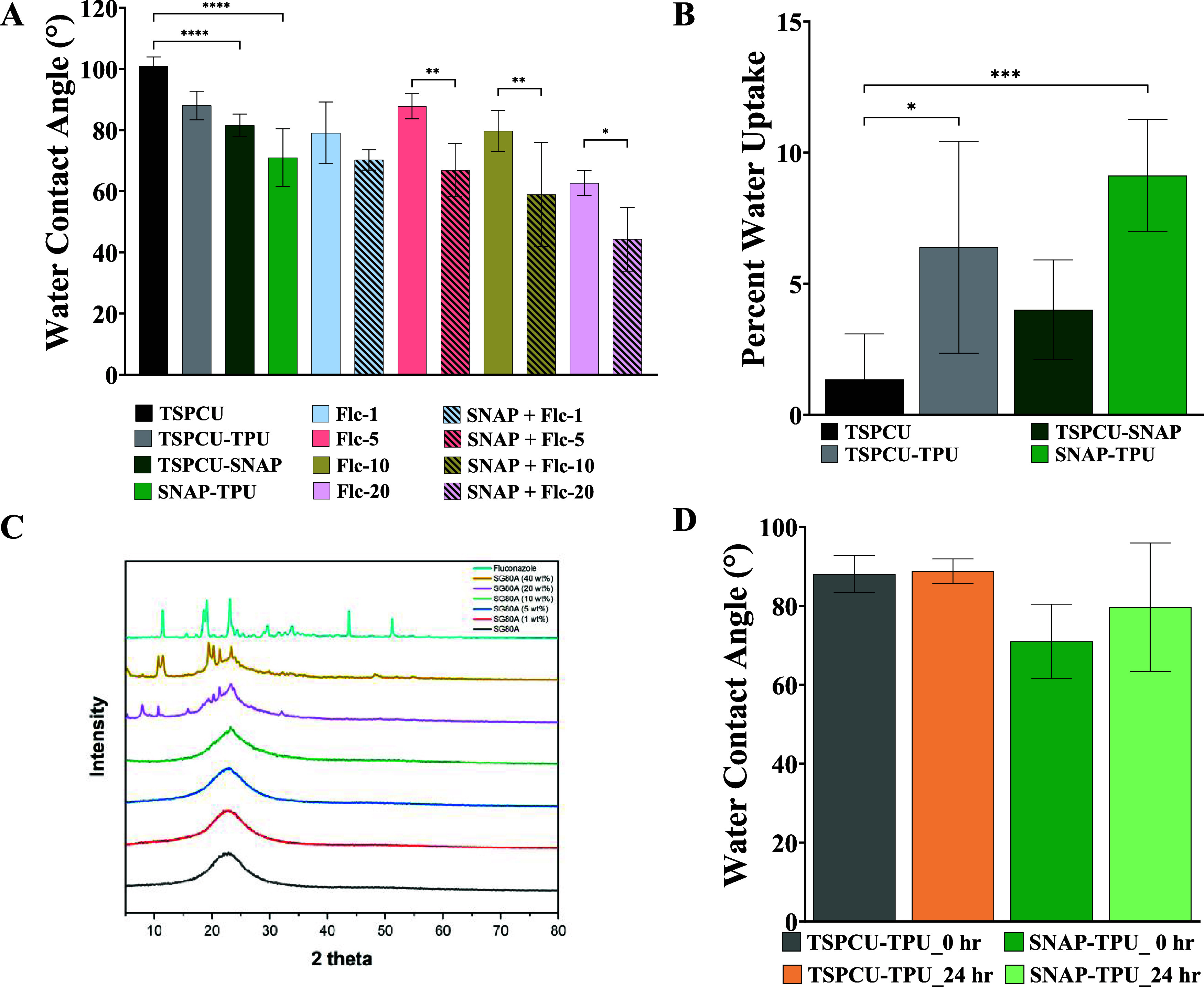
Characterization of the polymeric composites. (A) Surface wettability of the fabricated composites. WCA was measured using 10 μL of water. (B) Percent water uptake of TSPCU composites. (C) X-ray diffraction spectra of fluconazole incorporated TPU. (D) Coating stability studies for TSPCU + TPU composites over 24 h. Data represent mean ± standard deviation, *n* > 3. Statistical analysis: One-way ANOVA coupled with Tukey’s test; * indicates *p* < 0.05, ** indicates *p* < 0.001, *** indicates *p* < 0.0005, **** indicates *p* < 0.0001.

The surface wettability of a biomaterial is crucial in determining the interactions with the physiological environment. Moreover, higher hydrophilicity has also been related to lower biofouling and enhanced biocompatibility.^43,44^ The water contact angle (WCA) of the fabricated composites was measured to evaluate the surface wettability. Results from the WCA analysis are shown in Figure 1A. TSPCU substrates had a WCA of 101.0 ± 2.9° which is comparable to previously published reports.^45^ Top coat with high-water-uptake TPU reduced the WCA to 88.1 ± 4.7°. Blending of SNAP to TSPCU decreased the WCA to 81.5 ± 3.7°. In other reports, SNAP incorporation has been shown to increase surface wettability.^27^ This reduction in WCA has been attributed to microstructures formation on the polymeric surface and hydrogen bonding with SNAP molecules. Further coating of TPU on the TSPCU-SNAP composites reduced the WCA to 71.0 ± 9.4° demonstrating the slightly hydrophilic properties of TPU top coat. Incorporating fluconazole into the TPU interphase decreased the water contact angle in a dose-dependent manner. A similar decrease with fluconazole incorporation into polymeric matrix has been reported previously and can be attributed to the relative hydrophilicity of fluconazole.^46^ The lowest WCA was obtained with 20 wt % of fluconazole blended into the top coat (62.7 ± 4.1°). However, with the highest concentration of fluconazole (40 wt %), the WCA went up to 87.8 ± 4.1°. This increased WCA with >20 wt % of fluconazole could be attributed to the higher surface features originating from the reduced solubility of fluconazole in the polymeric matrix. These observations motivated use of 20 wt % as the highest concentration tested for biological evaluations.

Next, the changes in water uptake of the polymeric composites were evaluated. The TSPCU ChronoSil is a low-water-uptake polycarbonate, whereas TPU SG-80A has higher water uptake potential. TSPCU composites with and without SNAP were top-coated with TPU and the water uptake was measured after 24 h. The results shown in Figure 1B demonstrate that base TSPCU composites had a water uptake of <2% and matched published literature as well as the manufacturer’s specification.^45^ As expected, top coating with TPU significantly enhanced the water uptake to 6.4 ± 4.0%. The incorporation of SNAP into the TSPCU matrix increased the water uptake to 4.0 ± 1.9%. Further addition of TPU top coat on SNAP-containing TSPCU composites enhanced the water uptake to 9.1 ± 2.1%. These results corroborate the static WCA results; higher-water-uptake composites have higher surface wettability.

### 3.2 Attenuated Total Reflectance-Fourier Transform Infrared Spectroscopy

ATR-FTIR studies were performed to study the chemical composition of polymer composites containing SNAP and fluconazole. Resolution of characteristic peaks of SNAP including 3350 cm^–1^ (N–H stretching), 1680 cm^–1^ (C=O) stretching and 1497 cm^–1^ (N=O) stretching were observed with SNAP-TPU and SNAP + Fluconazole 20 wt % composites (Figure S2), which are consistent with prior findings.^47^ Additional peaks consistent with fluconazole including 3120 cm^–1^ (O–H stretching) and 1620 cm^–1^ (C=N stretching) were observed with fluconazole 20 wt % and SNAP + Fluconazole 20 wt % composites (Figure S2) and match with crystalline Fluconazole. Other characteristic peaks of polyurethane including aliphatic character (2936/2856 cm^–1^) and ether bonds (1109 cm^–1^) were observed.

### 3.3 X-ray Diffraction

To explore the loading behavior of fluconazole in the TPU coating, XRD was conducted demonstrating the changes in crystallinity on increasing wt % of fluconazole. Different wt % of fluconazole (1, 5, 10, 20, and 40) were analyzed with XRD and compared to the TPU control and fluconazole powder control. The TPU showed only one broad amorphous peak from 5 to 80 (2θ) which started narrowing at 10 wt % fluconazole incorporation, illustrating the increased crystallinity of the polymer coating. The 40 wt % samples were significantly more crystalline causing mechanical integrity to be compromised, and this result is similarly reflected in the XRD diffraction patterns. The spectra obtained are shown in Figure 1C. Additionally, the percent crystallinity was calculated using OriginPro software by determining the area of the crystalline peaks and dividing that by the total area of the XRD spectra (Table S2). As more fluconazole is added to the polymer composite, there was an increase in the % crystallinity of the composites. The triclinic structure of fluconazole became more apparent as the weight percentage of fluconazole is increased in the polymeric matrix as evidenced by peaks at 2θ 11.42, 19.08, and 23.12.

### 3.4 Coating Stability

Polymeric composites with different water uptake and surface wettability can sometimes be noncompatible and may delaminate over time. There are no reports published with a combination of TSPCU ChronoSil and TPU SG-80A; hence, we evaluated the stability of the TPU top coat on TSPCU base substrates. As discussed above in Section 3.1, there was a difference between WCA and water uptake of TSPCU and TPU and a change in WCA over time can provide insight into the stability of the top coat. The results summarized in Figure 1D show negligible change in the WCA of top-coated composites with or without SNAP over 24 h. These data support the compatibility of these two polymers.

### 3.5 Surface Morphology Analysis

**Figure 2:**
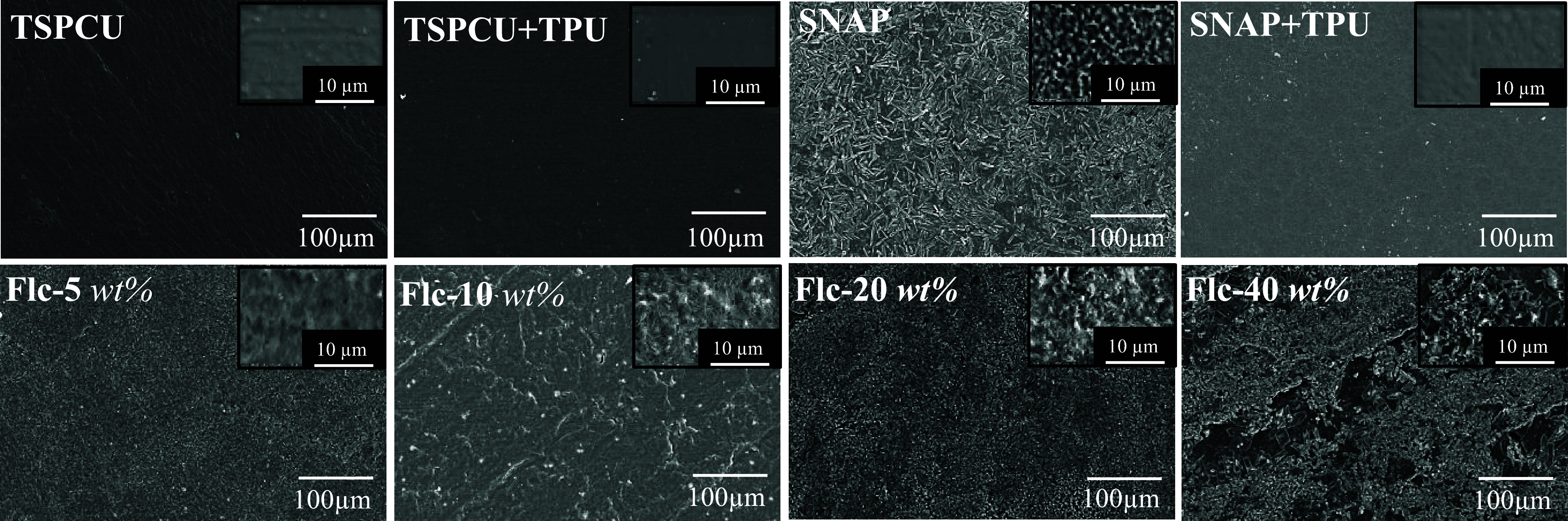
Representative scanning electron microscopy of the polymeric substrates showing surface morphology. Scale bar: 100 μm, inset images 10 μm.

The surface morphology of indwelling devices can significantly alter the surface wettability, biofouling, and functionality of the device. The topography of the samples was studied using a scanning electron microscope. As shown in Figure 2, the TSPCU composites had a smooth surface with minimal topographical features. Top coat with TPU further smoothened the surface. The incorporation of SNAP into the TSPCU composite had a rough surface resulting from the SNAP crystalline aggregates that formed near the polymer surface. A top coat of TPU on the SNAP-incorporated composites resulted in a smoother surface with a similar appearance to the TPU-coated TSPCU controls. Further incorporation of fluconazole into the TPU top coat imparted surface features with higher concentrations showing higher surface texturing. However, the distribution of fluconazole remained uniform until 20 wt % fluconazole concentration. The 40 wt % fluconazole coating resulted in uneven coating with visible patches.

Elemental mapping of a cross section of the fabricated composites was performed to show the presence of SNAP and fluconazole in the polymeric layers. The results summarized in Figure S3 demonstrate the presence of SNAP by the emergence of sulfur in the SNAP-containing composites. Fluconazole was shown by the presence of fluorine in the polymeric matrix incorporated with fluconazole.

### 3.6 Fluconazole Release

**Figure 3:**
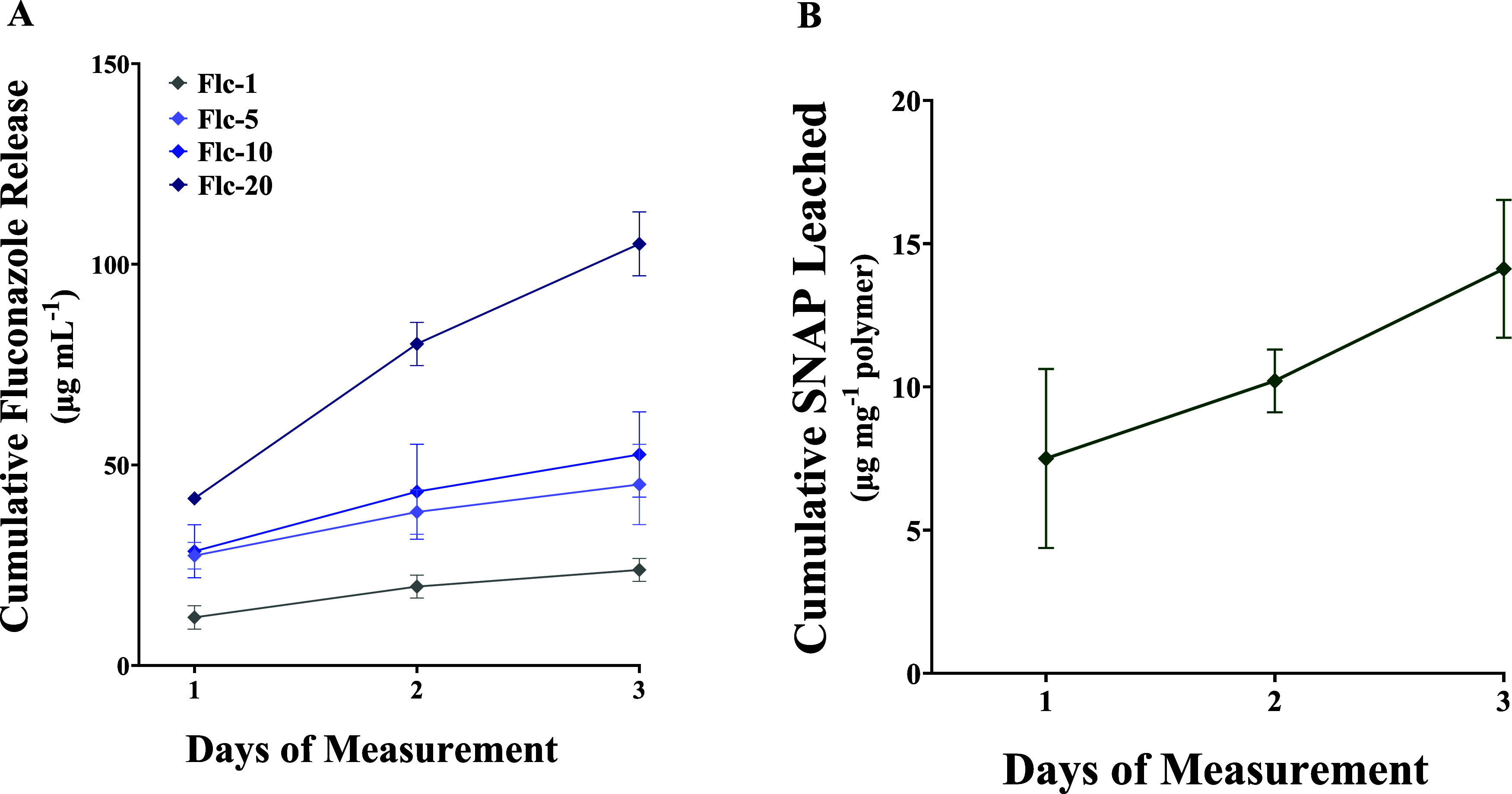
Fluconazole and SNAP release from SNAP-Fluconazole composites. Cumulative release of (A) fluconazole, (B) SNAP, and SNAP-fluconazole composites over 3 days under physiological conditions (37 °C, pH 7.4, submerged in PBS-EDTA). Data represent mean ± standard deviation, *n* > 3.

Release of fluconazole from the polymeric matrix is essential for reducing fungal infection in the physiological environment. The amount of fluconazole diffused into the surroundings under physiological conditions over 72 h was evaluated at absorbance 203 nm. The molar absorption coefficient of fluconazole was found to be 13.43 L mol^–1^ cm^–1^ at 203 nm. The cumulative and daily release of fluconazole from the polymeric samples is shown in Figure 3A and Table S3. There was a dose-dependent increase in the total amount of fluconazole diffused at each time point tested, with 1 wt % composite releasing the lowest and 20 wt % the highest levels of fluconazole. After 72 h, composites dip-coated with 20 wt % fluconazole had a cumulative release of 105.1 ± 8.0 μg mL^–1^ of fluconazole, whereas the 1 wt % composites released 23.8 ± 2.9 μg mL^–1^. The amount of fluconazole released was directly proportional to the amount of fluconazole incorporated into the matrix, indicating the potential to fine-tune the release as required.

From further kinetic analysis, it is shown that lower weight percents of fluconazole exhibited enhanced cumulative fractions of release through 8 h (Figure S5A), while the higher weight percent formulations such as 20 wt % released proportionally lower amounts of the loaded drug. This was corroborated by fits of the diffusion kinetics to Fick’s second law of diffusion, showing that the 1 wt % fluconazole formulation exhibited significantly enhanced diffusion rates and release of the fluconazole payload compared to all other formulations (Figure S5B). No significant differences in diffusion rates were observed with the higher weight percentage fluconazole formulations, suggesting enhanced crystallization of the cargo within the polymeric backbone and reduced levels of release with respect to total payload.

These amounts are much higher than past reports with fluconazole-releasing materials.^46,48,49^ This can be attributed to the unique design of this biomaterial with a high-water uptake TPU allowing rapid diffusion of fluconazole from the matrix. Moreover, the MIC_50_ of fluconazole against various lab and clinical *Candida* spp. has been reported to be 0.06 to 15 μg mL^–1^,^46^ similar to the amounts released from the fabricated composites. The growth curve of *C. albicans* treated with fluconazole is given in Figure S7. These results support the antifungal potential of the fabricated composites and were further evaluated *in vitro* for antibacterial capabilities.

### 3.7 SNAP Diffusion

A characteristic essential for NO-based biomaterials’ success is preserving the NO donor inside the polymeric matrix. Uncontrolled or rapid diffusion of the NO donor from the films can exhaust the reservoir quickly leading to depletion of NO-releasing capabilities. Additionally, high concentrations of SNAP released by the biomaterial can elicit undesired complications such as cytotoxicity. SNAP diffusion on the surface is also influenced by the surface wettability of the polymer with high hydrophilicity polymers leaching SNAP at a faster rate than their hydrophobic counterparts. Since the NO blended hydrophobic TSPCU was top-coated with relatively hydrophilic TPU, the SNAP diffusion from the composites should be evaluated. The cumulative and daily release of SNAP from the polymeric matrix is shown in Figure 3B and Table S3.

The molar absorption coefficient of SNAP was found to be 756.88 L mol^–1^ cm^–1^ at 340 nm. The composites did not diffuse a significant amount of SNAP under physiological conditions, and the amount was under 10 μg mg^–1^ of the polymeric mass. Incorporating TPU with fluconazole had trivial effects on SNAP diffusion from the composites. The amount diffused was <10% of the total mass of blended SNAP indicating the high stability of the NO donor in the polymeric matrix. A similar percentage of SNAP diffusion from hydrophobic polymers has been reported in the literature.^35,50^ These results show the potential of long-term applications of these composites without depleting the NO donor reservoir.

### 3.8 Nitric Oxide Release Evaluation

**Figure 4:**
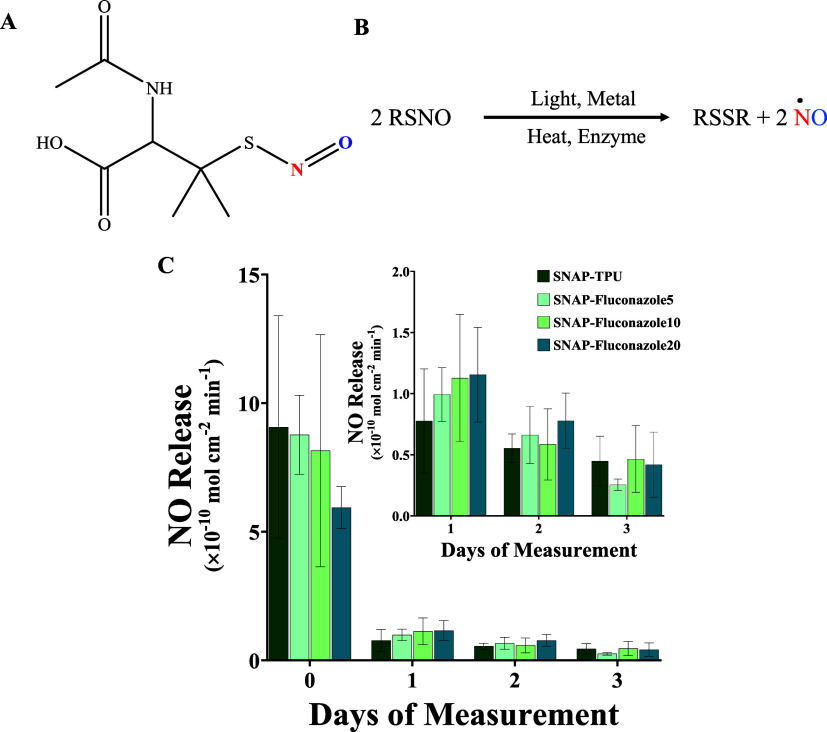
Real-time NO release kinetics from SNAP + fluconazole composites. (A) Structure of NO donor *S*-nitroso-*N*-acetylpenicillamine (SNAP). (B) Schematic of NO release from SNAP catalyzed by light, heat, enzymes, and metal ions. (C) Prolonged NO release from SNAP + fluconazole composites under physiological conditions (37 °C, pH 7.4, submerged in PBS). Data represent mean ± standard deviation, *n* > 3.

NO-releasing biomaterials have garnered interest as antibacterial, antithrombotic, and biocompatible alternatives to other polymeric biomedical devices. NO functionality is highly dose-dependent, and it is crucial to have a controlled release to induce desired bioactivity. Moreover, uncontrolled or burst release of NO from a biomaterial interface may lead to exhaustion of the donor reservoir hampering the longevity of the device. *S*-Nitrosothiols such as SNAP can be catalyzed by heat, light, metal ions, and moisture to evolve NO.^51,52^ Endothelial levels of NO have been described as a surface flux between 0.5 and 4 × 10^–10^ mol cm^–2^ min^–1^ and are accepted as the physiological level of NO.^53^ NO donor SNAP was physically blended into TSPCU, followed by polymeric composite fabrication using the solvent casting method. The SNAP-containing polymeric composites were further dip-coated with high-water-uptake TPU with and without fluconazole to yield the final composites. Real-time NO released from the polymeric composites under physiological conditions (37 °C, pH 7.4) was recorded using a chemiluminescent-based NOA. Special care was taken to ensure the reaction system was free of other NO-catalyzing agents, such as metal ions, enzymes, and light. The results from NO release profiling over 72 h are shown in Figure 4. SNAP composites coated with TPU showed the highest NO flux on day 0 with 9.07 ± 4.33 × 10^–10^ mol cm^–2^ min^–1^, which reduced to 0.78 ± 0.43 × 10^–10^ mol cm^–2^ min^–1^ after 24 h. The NO release was maintained at physiological levels for 72 h (0.45 ± 0.20 × 10^–10^ mol cm^–2^ min^–1^). Adding fluconazole into the TPU top coat slightly reduced the day 0 NO flux yielding 8.77 ± 1.54 × 10^–10^ mol cm^–2^ min^–1^ of NO for 5 wt % fluconazole composites. The lowest day 0 release was obtained with fluconazole 20 wt % composites (5.94 ± 0.81 × 10^–10^ mol cm^–2^ min^–1^).

Fluconazole 20 wt % composites had slightly lower water uptake than the other concentrations (Table S1), which could be responsible for the slightly decreased NO flux on day 0. High-water-uptake matrices allow PBS to penetrate the matrix and contact the NO donor reservoir, hastening NO release. Similar observations of surfaces with higher water uptake and surface wettability rapidly releasing NO have been reported in the literature.^45^ These highly hydrophilic materials end up exhausting the NO reservoir in the short term (mostly <24 h);^37,45,54^ however, the composites reported here could release NO for at least 72 h. All of the composite types except fluconazole 5 wt % could release physiological levels of NO over 72 h. Fluconazole 5 wt % released 0.25 ± 0.05 × 10^–10^ mol cm^–2^ min^–1^ of NO at the end of 72 h. Although the levels are lower than physiological conditions, these levels of NO have been shown to have antibacterial properties *in vitro*.^55,56^

It is hypothesized that since the high-water-uptake TPU was only employed as a thin top coat it could not facilitate much water penetration into the base TSPCU. Moreover, fluconazole is poorly soluble in water and its increasing concentration directly prevents water absorption as shown by the water uptake results (Table S1). The fluconazole-containing-TPU layer, despite the enhanced surface wettability, acts as a physical barrier preventing PBS penetration to the TSPCU matrix. Furthermore, bacterial infections and biofilm formation can occur within the first few hours of a device application. The higher NO release on day 0 could rapidly kill any planktonic bacteria contacting the device. The release of NO over 72 h from the composites can prevent any infection possible on the device surface.

### 3.9 Antifungal Efficacy

*C. albicans*-mediated infections have increased significantly in the past decade and cause >1.5 million deaths globally every year.^57^ According to a survey of healthcare-associated infections in the United States, *Candida* species were the major cause of bloodstream infections closely followed by coagulase-negative staphylococcus species.^58^ The development of antifungal biomaterials has not received as much attention as their antibacterial counterparts leaving a gap that needs to be filled. Furthermore, in clinical settings, antibiotics are administered as the first line of treatment upon any infection. The focus on bacterial infections leads to fungal infections being overlooked and worsens over time.^6^ Hence, it is imperative to focus on designing biomaterials that can innately resist fungal infections and bacterial infections. Additionally, drug-eluting medical devices offer controlled, and localized release of antifungal agents at the potential site of infection precluding systemic drug administration.^48^ Soriano et al. developed fluconazole-releasing implantable rods for osteomyelitis therapy and demonstrated excellent antifungal and drug-releasing properties *in vitro* and *in vivo*.^49^ Another recent report by Toirac et al. showed that fluconazole-loaded coatings could prevent localized fungal infection in titanium-based implants.^48^ This project combined antifungal fluconazole release with antibacterial NO release to fabricate a broad-spectrum antimicrobial biomaterial.

Fluconazole is a bis-tri-azolefluorine-substituted antifungal agent with 90% bioavailability.^8,49^ It is an FDA-approved drug with multiple clinical trials demonstrating its efficacy against various fungal strains.^10,59^ Moreover, the amount of fluconazole released from this fabricated material is significantly lower than the systemic dosage deemed safe in patients, indicating the tolerability of the material.^60^ However, the highly localized drug delivery would ensure its efficacy in preventing fungal infection at lower payloads.

**Figure 5:**
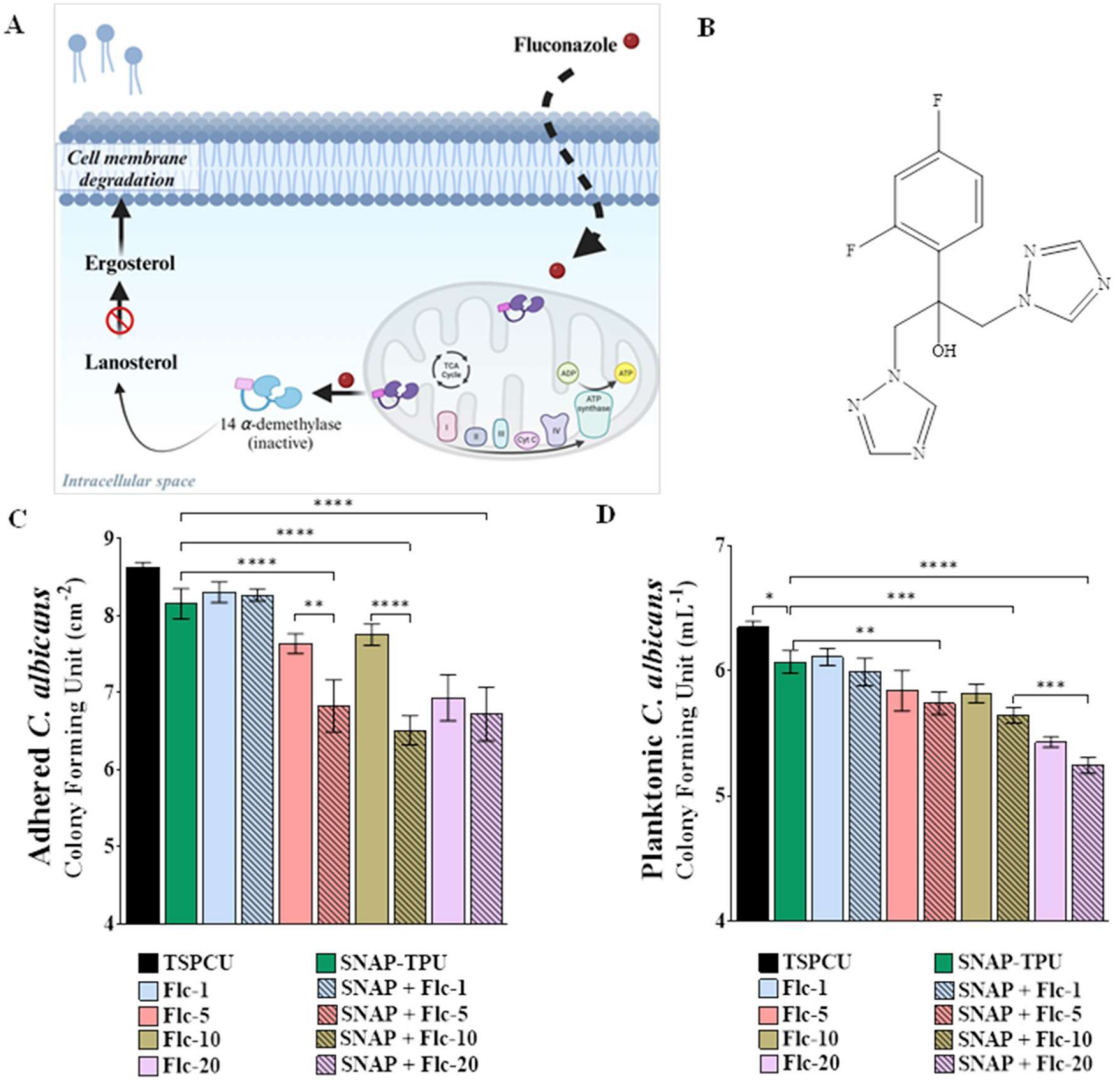
Antifungal evaluation of SNAP-fluconazole composites. (A) Mechanism of fluconazole antifungal action. Fluconazole inactivates cytochrome P_450_ enzyme 14 α-demethylase which inhibits synthesis of cell membrane component ergosterol from lanosterol, causing membrane degradation and cell death. (B) Structure of the drug fluconazole. (C) Reduction in adhered *C. albicans* after 24 h under physiological conditions. (D) Reduction in planktonic *C. albicans* after 24 h under physiological conditions (37 °C, pH 7.4, PBS). Data represent mean ± standard deviation, *n* = 3. Statistical analysis: One-way ANOVA coupled with Tukey’s test; * indicates statistical significance of *p* < 0.05, ** indicates *p* < 0.01, *** indicates *p* < 0.001, and **** indicates *p* < 0.0001.

The antifungal effects of the fabricated material against *C. albicans* were evaluated following standard ASTM E2180 protocol with minor modifications previously reported.^27,61^ The antifungal results for both adhered and planktonic fungi are summarized in Figure 5 and Table S4. As anticipated, SNAP had moderate effects on fungal viability in both adhered and planktonic forms (64.3 ± 12.4 and 46.3 ± 9.7%) compared to controls. These results align with our previous reports on the antifungal effects of NO-releasing material.^18^ These observations are also reflected in the growth curve of *C. albicans* treated with SNAP, as shown in Figure S8. Incorporating fluconazole into the polymer matrix revealed a concentration-dependent increase in antifungal activity in adhered and planktonic forms. Composites containing 20 wt % fluconazole reduced viable fungi by 97.7 ± 1.1 and 87.9 ± 1.0% in adhered and planktonic forms, respectively, compared to TSPCU controls. The lowest activity was, of course, obtained with 1 wt % fluconazole composites showing 51.2 ± 15.6 and 41.8 ± 7.3% in adhered and planktonic forms, respectively.

The concentration-dependent response could be explained by the higher release of fluconazole from the higher-concentration composites under physiological conditions. Minimal difference was observed between 5 and 10 wt % fluconazole, which was expected by the similar fluconazole release profile between the two sample types.

The addition of NO release via SNAP blending enhanced the antifungal activity of the composites even though NO by itself only had moderate reductions. These results are supported by previously published reports demonstrating the synergistic activity of NO donors with azole-based antifungals in eradicating fungal infections.^5,30^ All of the SNAP + fluconazole composites showed >95% reduction in adhered fungi after 24 h and were not statistically significantly different from each other. Similarly, fungal killing was observed in the planktonic forms where the highest fluconazole concentration composites (20 wt %) reduced the viability by 92.1 ± 0.9% compared to controls. 5 and 10 wt % composites showed a moderate reduction between 75 and 80%. These results demonstrated the excellent antifungal potential of the fluconazole and SNAP-containing composites against *C. albicans*, except for 1 wt % fluconazole composites, and were tested further for antibacterial capabilities *in vitro*.

### 3.10 Antibacterial Efficacy

**Figure 6:**
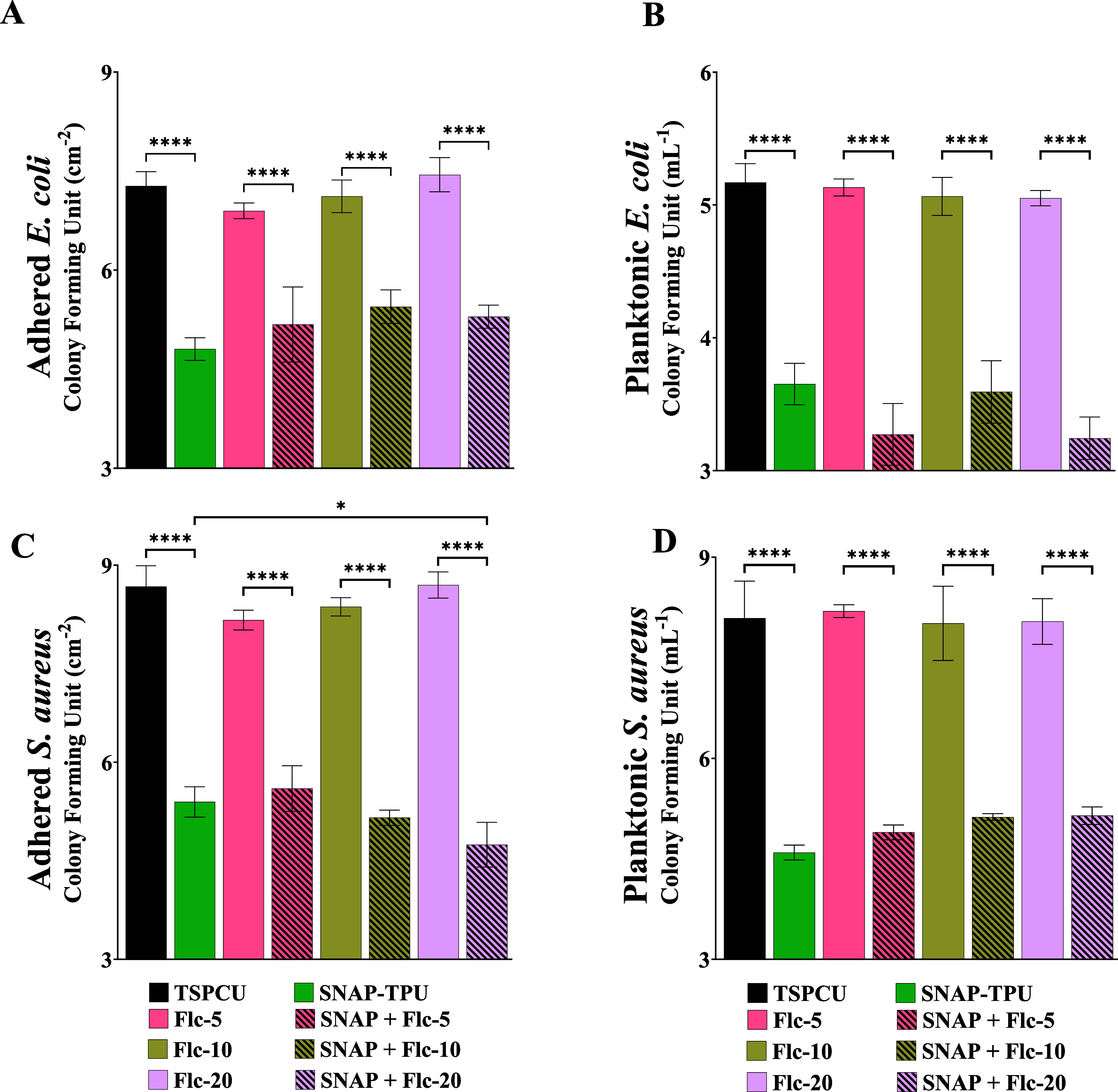
Antibacterial evaluation of SNAP-fluconazole composites. Reduction in viable bacteria after 24 h under physiological conditions (37 °C, pH 7.4, PBS) (A) adhered *E. coli*, (B) planktonic *E. coli*, (C) adhered *S. aureus*, and (D) planktonic *S. aureus*. Data presents mean ± standard deviation, *n* = 3. Statistical analysis: One-way ANOVA coupled with Tukey’s test; * indicates statistical significance of *p* < 0.05, ** indicates *p* < 0.01, *** indicates *p* < 0.001, and **** indicates *p* < 0.0001.

NO-releasing materials have demonstrated excellent antibacterial efficacy against a variety of clinically relevant Gram-positive and Gram-negative strains.^24,25^ We selected Gram-positive catalase- and coagulase-positive, *S. aureus* and oxidase-negative, Gram-negative *E. coli* as model organisms to demonstrate the broad-spectrum antibacterial activity of the fabricated composites. These two strains are common in hospital-acquired infections (HAIs) and are responsible for ∼20% of all HAIs.^62^ Additionally, *E. coli* is probably the biggest cause of urinary tract infections, whereas *S. aureus* and *C. albicans* are the major contributors to bloodstream infections.^58^ The antibacterial efficacy of the SNAP-fluconazole material was evaluated against these two strains following ASTM standardized E2180 protocol^61^ with minor modifications, as explained in Section 2.7. The result from the antibacterial assessment is summarized in Figure 6 and Table S5. Since 1 wt % fluconazole composites did not show potent antifungal properties, they were not evaluated in the antibacterial studies. SNAP showed significant efficacy in reducing the viable bacterial load in both *E. coli* and *S. aureus* (>2 and >3-log reduction, respectively, compared to TSPCU controls) in adhered and planktonic forms. These results were expected as NO-releasing materials have routinely shown such bioactivity in the literature with similar NO payloads.^63−65^ Incorporation of only fluconazole had insignificant effects on planktonic bacteria, which was anticipated as fluconazole has shown to be ineffective against bacterial strains without cytochrome P_450_, such as *E. coli*.^66^ Although fluconazole has shown moderate effectiveness against other bacterial strains and can enhance the efficacy of other antibacterial agents.

A combination of SNAP and fluconazole showed excellent reduction both in planktonic and adhered bacteria, comparable to TSPCU. The composites performed better against *S. aureus* (>3-log reduction) than *E. coli* (>1.8-log reduction). SNAP + Fluconazole 20 wt % composites significantly outperformed SNAP-blended composites in reducing the adhesion of *S. aureus*. This can be attributed to the high surface wettability of the SNAP + Fluconazole 20 wt % composites. Polymeric material with high surface wettability can prevent bacterial adhesion on the surface.^43,67^ Protein adsorption is the first step in biofouling and is favored by hydrophobic surfaces with low wettability and high surface energy. A hydrophilic biomaterial allows the formation of a hydration layer on the surface along with low surface energy, essentially repelling any biofoulants (proteins, cells, microbes) from attaching to the surface.^43,67^ The slight bias toward efficiently killing Gram-positive *S. aureus* by NO-releasing biomaterials has been reported previously.^27,41^

Overall, the SNAP-fluconazole composites showed promising antibacterial activity toward the clinically relevant Gram-positive and Gram-negative strains tested. The addition of nonantibacterial fluconazole significantly affected the antibacterial potential of the composites at the tested formulations.

### 3.11 Cytocompatibility

Cytocompatibility is an essential aspect of any biomedical device, and a lack thereof can make the device unusable. The combination of SNAP and fluconazole used in this study is not expected to elicit cytotoxicity toward mammalian cells. The components used in this study are biocompatible, and the doses released into the physiological environment are well within tolerable range.^25,60^ We evaluated the cytotoxicity of the fabricated composites following the protocols recommended by the International Standards Organization (ISO) with minor modifications.^42^ Mouse fibroblast cells (NIH-3T3) were used as model cell lines for this study. These cell lines are the most common cell types used for biomaterial cytotoxicity evaluation.

**Figure 7:**
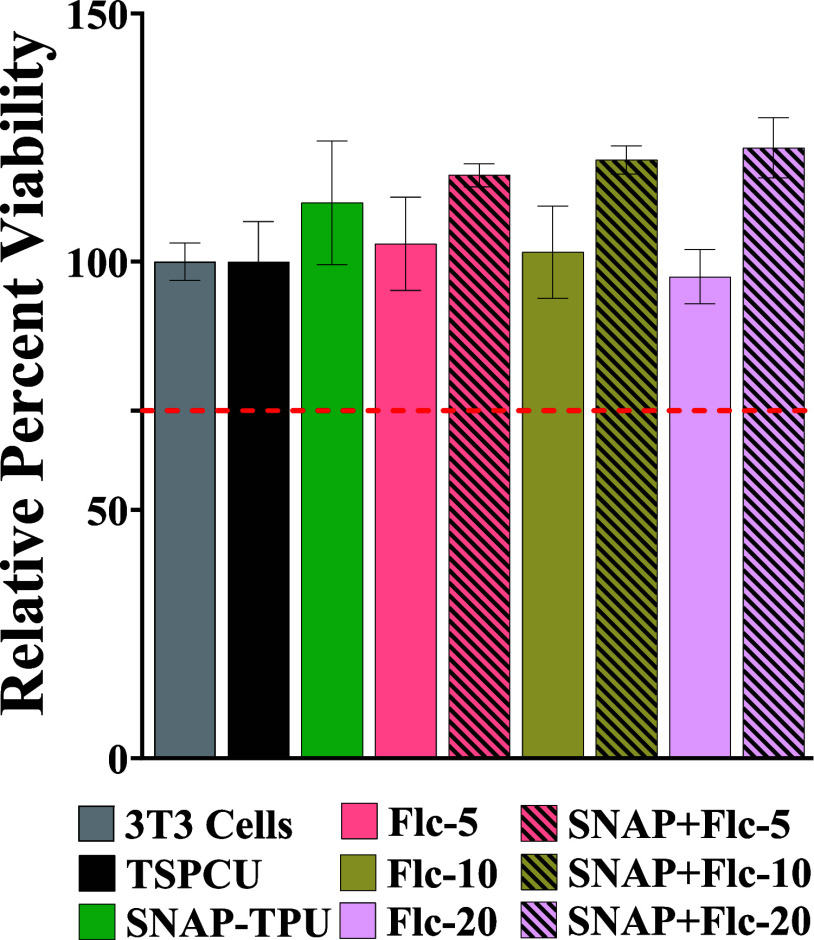
Cytocompatibility evaluation of the SNAP-fluconazole composites. 3T3 cells exposed to leachates for 24 h under physiological conditions followed by viability estimation using CCK-8 kit. Data represent mean ± standard deviation, *n* = 5.

The ISO standards define a biomaterial to be cytotoxic if it causes >30% reduction of viability in mammalian cells. The results summarized in Figure 7 demonstrate that the composites were cytocompatible with mouse fibroblast cells; all m had a viability of >95% compared to untreated cells. Moreover, SNAP-containing composites showed a slight increase in percentage viability compared to untreated cells. This can be attributed to the proliferative effects of NO on fibroblast cells, a key aspect involved in wound healing.^68^ Overall, the presented biomaterial with dual-action antibacterial and antifungal potential was cytocompatible with mouse fibroblast cells and is suitable for further evaluation.

## 4 Conclusions

This study reports a novel biomaterial with codelivery of NO and fluconazole intended to prevent bacterial as well as fungal infections on biomedical devices used in clinical settings. The design was achieved by fabricating SNAP-blended TSPCU composites followed by facile dip-coating with high-water-uptake TPU blended with fluconazole. The resulting composites showed hydrophilic surface properties suggesting the antifouling potential of the biomaterial. The polymeric composites released physiological levels of NO (>0.5 × 10^–10^ mol cm^–2^ min^–1^) for at least 72 h while preserving the SNAP donor reservoir inside the polymeric matrix. The composites could also release fungicidal levels of fluconazole (>45 μg mL^–1^ cumulative release) for 72 h under physiological conditions. Biological evaluations of the material revealed antibacterial and antifungal properties against clinically relevant *S. aureus*, *E. coli*, and *C. albicans*, tested under physiological conditions for 24 h. A 1- to 2-log reduction of fungal load in adhered and planktonic *C. albicans* was obtained, whereas a 3 to 4-log reduction for *S. aureus* and 1.6- to 2.1-log reduction in *E. coli* were obtained. The material killed bacteria and fungus both in surface-adhered and planktonic forms, demonstrating the broad-spectrum anti-infective capabilities. Furthermore, the composites showed no cytotoxic behavior against mouse fibroblast cells when tested *in vitro* endorsing the cytocompatibility of the novel biomaterial. Overall, this study presents a novel strategy for designing anti-infective biomaterials to prevent infection on a device surface, an otherwise critical complication in clinical settings. The long-term application potential, scalability, and cost efficacy of this material require further evaluation before considering it for translating into a commercial product.

## Supporting Information Available

Raman spectra of fluconazole, ATR-FTIR spectra, scanning electron microscopy coupled with energy-dispersive X-ray spectroscopy, calibration curves, fluconazole diffusion kinetics, *C. albicans* growth curves treated with fluconazole or SNAP, water uptake of fluconazole incorporated composites, crystallinity quantification, SNAP and fluconazole release, and antimicrobial activity of the composites (PDF)
