# Supplementary material for: Combating Concomitant Bacterial and Fungal Infections via Codelivery of Nitric Oxide and Fluconazole
Source: ACS Appl Mater Interfaces. 2025 Apr 14;17(16):23613–26. doi: 10.1021/acsami.5c00174 (PMC12022954; doi:10.1021/acsami.5c00174)
Supplement: Supplementary file 1 — am5c00174_si_001.pdf [file am5c00174_si_001.pdf]

SUPPORTING INFORMATION

Combating Concomitant Bacterial and Fungal Infections *via* Co-Delivery of Nitric Oxide and Fluconazole

Rashmi Pandey, [a] *Natalie Crutchfield,* [a] *Mark Richard Stephen Garren,* [a] *Ekaa Manohar Kasetty,* [b] *Manjyot Kaur Chug,* [a] *Elizabeth J. Brisbois,* [a] *Hitesh Handa* [a, c] *

[a] School of Chemical, Materials, and Biomedical Engineering, College of Engineering, University of Georgia, Georgia 30602, United States

[b] Franklin College of Arts and Science, University of Georgia, Athens, Georgia 30602, United States

[c] Pharmaceutical and Biomedical Science Department, College of Pharmacy, University of Georgia, Athens, Georgia 30602, United States

*Corresponding Author: Dr. Hitesh Handa

Associate Professor

University of Georgia

iSTEM-2, 302 East Campus Rd

Athens, GA 30602

Telephone: (706) 542-8109

E-mail: hhanda@uga.edu

Supporting Figures

Raman Spectroscopy

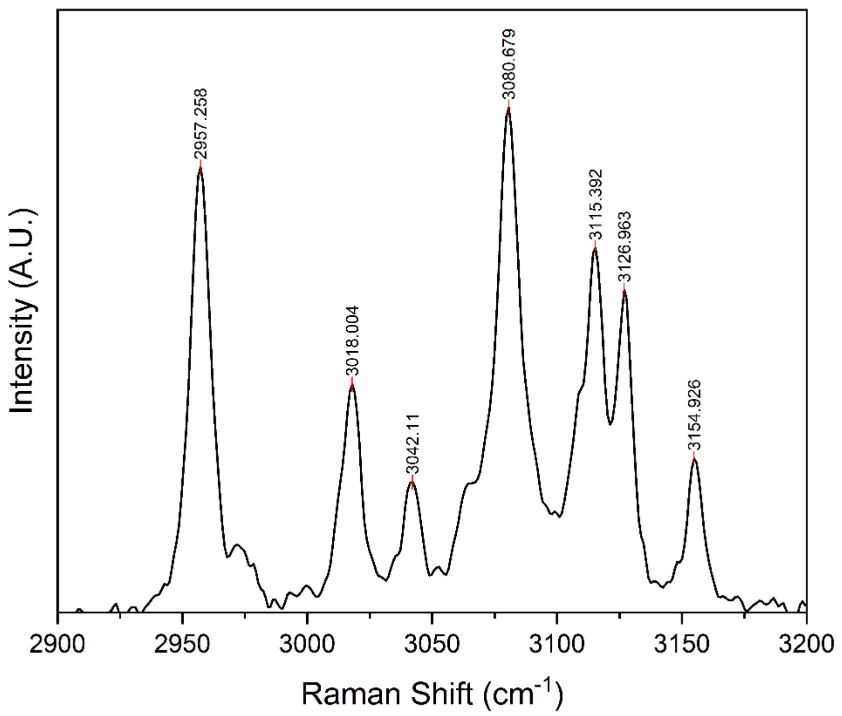

Figure S1: Raman Spectroscopy was completed using a Thermo Scientific DXR Raman microscope using 8 mW power at 532 nm from 2900 – 3200 cm^{-1} of commercially procured fluconazole powder. The 2975 peak for CH_2 stretch on the propane backbone as seen in the monohydrate form of fluconazole is not present supporting the XRD data to determine it is mostly present in the anhydrous polymorph form.

The spectra obtained matched published data for the polymorph I of fluconazole. [1]

Attenuated Total Reflectance-Fourier Transform Infrared Spectroscopy (ATR-FTIR)

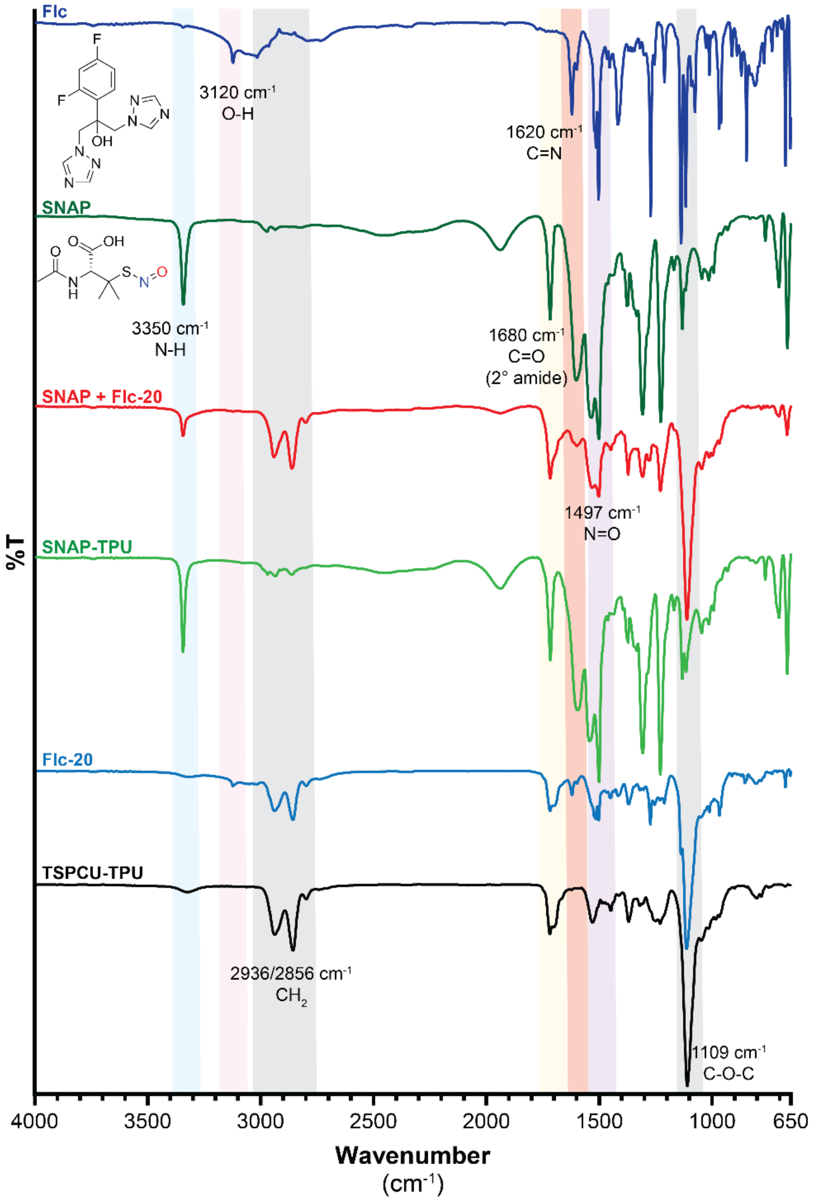

Figure S2: Attenuated total reflectance-Fourier transform infrared spectroscopy (ATR-FTIR) of SNAP and Fluconazole composites prepared with TSPCU and TPU compared against SNAP and Fluconazole powder.

Scanning Electron Microscopy coupled with energy dispersive X-ray spectroscopy (SEM/EDX)

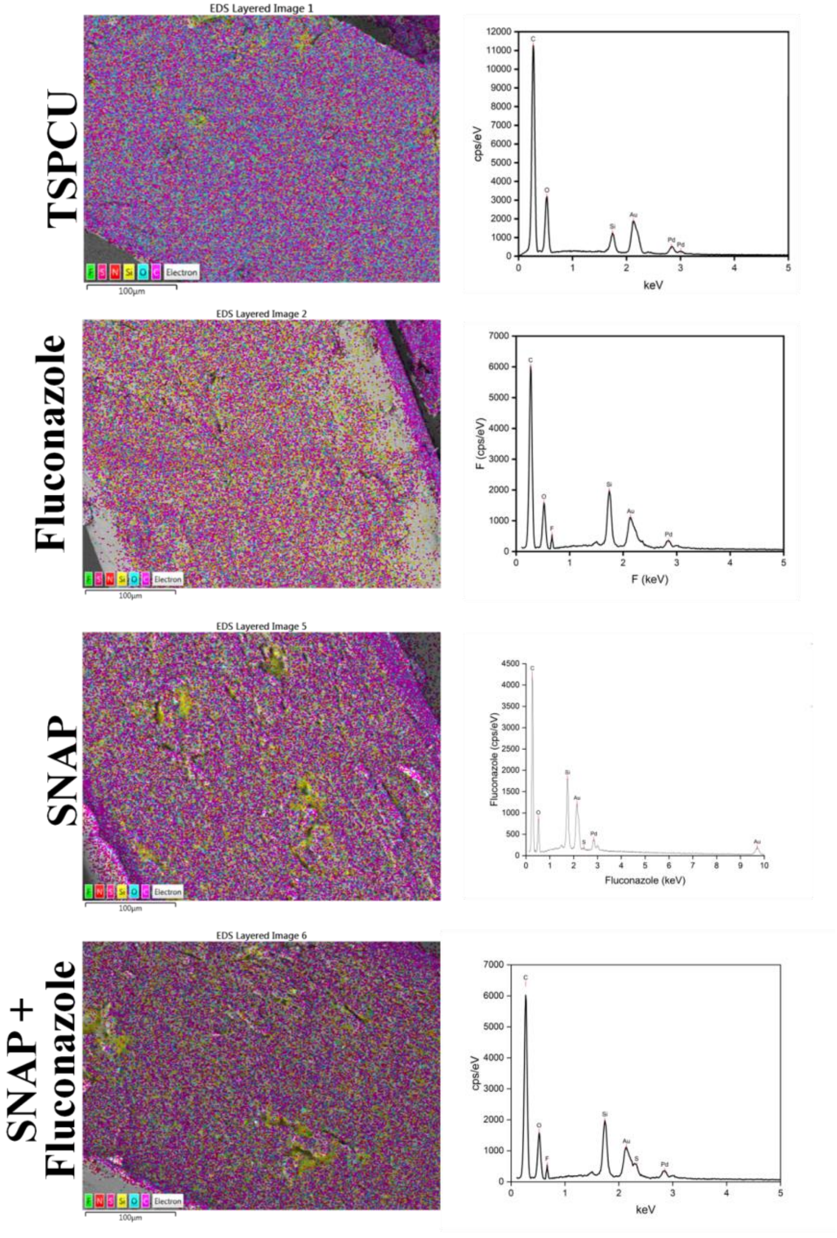

Figure S3: Elemental mapping of cross-sectional composites demonstrating the presence of SNAP (sulfur) and fluconazole (fluorine) in the polymeric matrix.

Fluconazole Calibration Curve and Absorption Spectra

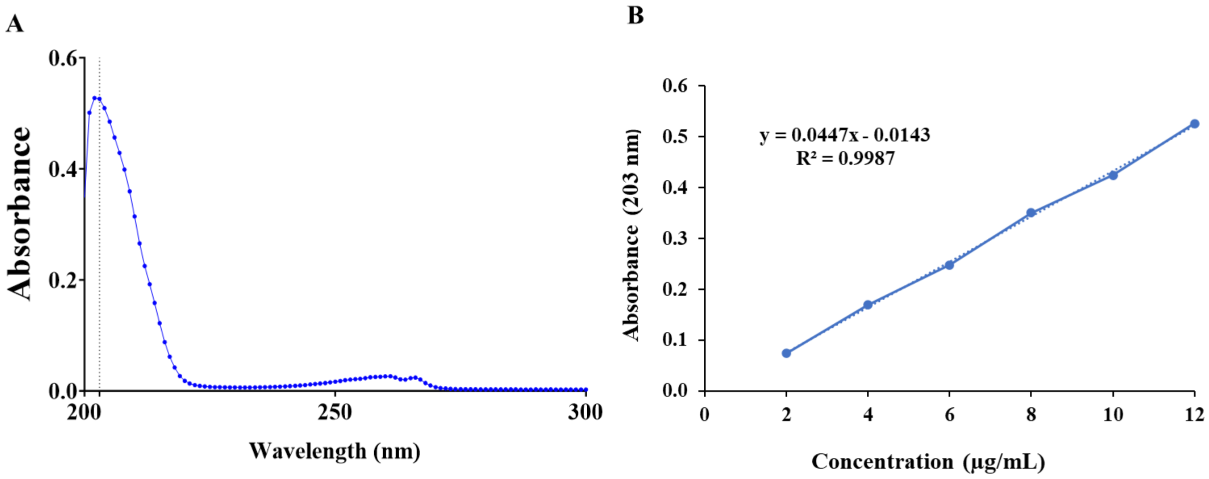

Figure S4: (A) Absorption spectra of fluconazole (B) Fluconazole calibration curve (in PBS). Fluconazole standards were prepared in PBS (pH 7.4) with concentrations ranging between 12 to 0 μg mL^{-1}. PBS was used as a blank. The absorbance was measured at 203 nm using a UV-Vis Spectrophotometer (Agilent).

Fluconazole Diffusion Kinetics Studies

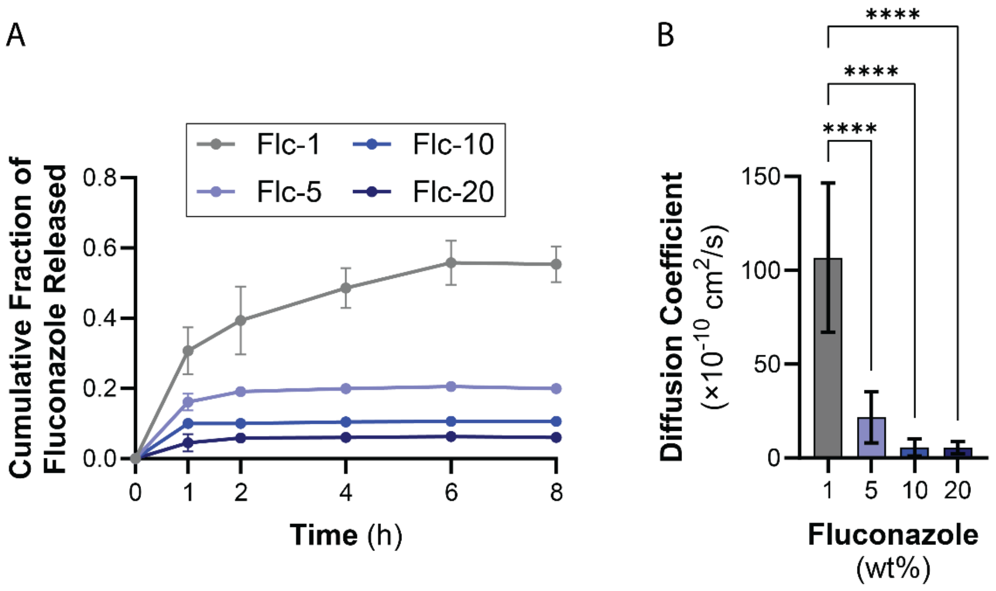

Figure S5: Fluconazole diffusion studies for determination of A) fractional release and experimental determination of B) diffusion coefficients (D) with diffusion-controlled model assuming validity of Fick's second law of diffusion with the various fluconazole formulations. Data shown as mean ± standard deviation ($n \geq 4$ per timepoint). Statistical significance defined as $p < 0.05$.

SNAP Calibration Curve and Absorption Spectra

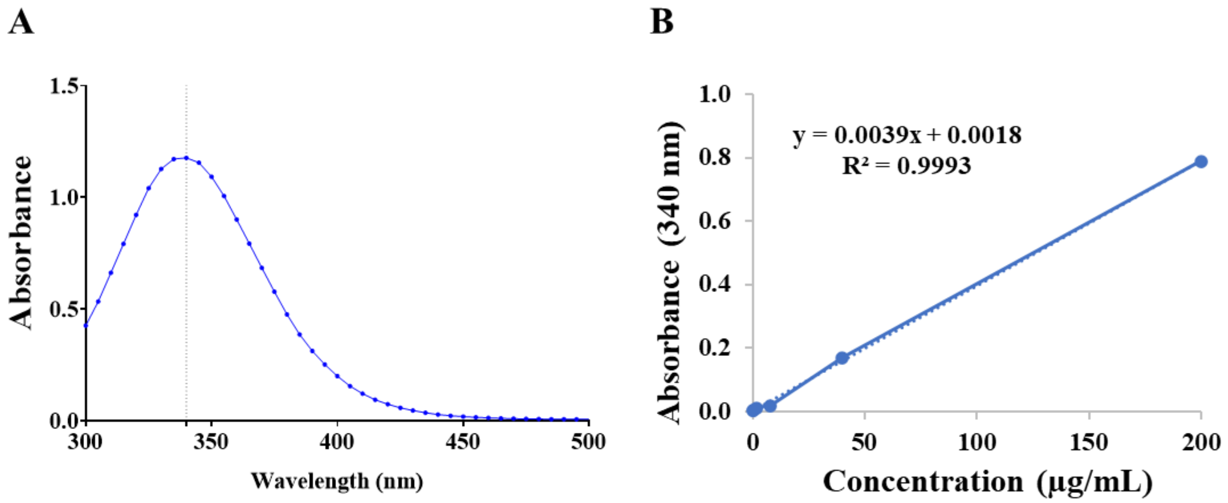

Figure S6: (A) Absorption spectra of SNAP (B) SNAP calibration curve (in PBS). SNAP was dissolved in PBS and the calibration curve was made ranging between 200 to 0 µg mL^{-1}. The absorbance was measured at 340 nm. The curve was used to extrapolate values of SNAP leached from the polymeric matrix under physiological conditions.

Growth Curve of Fluconazole Against *C. albicans*

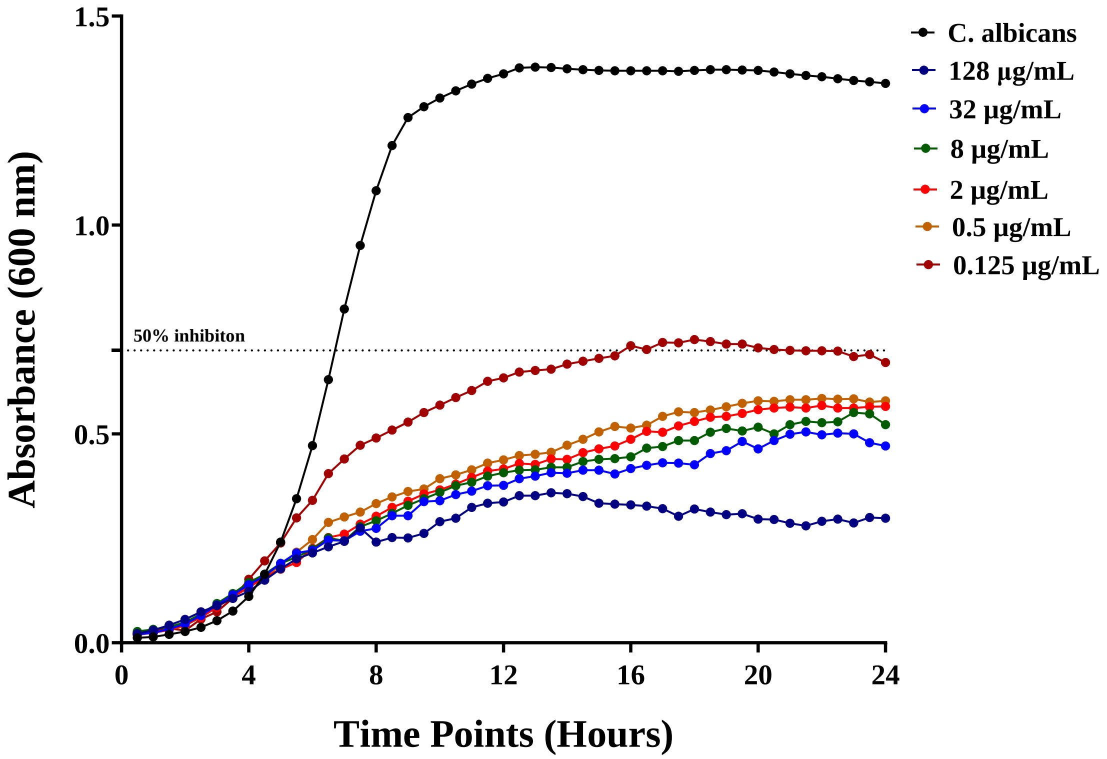

Figure S7: Growth curve of *C. albicans* treated with fluconazole. *C. albicans* was cultured in yeast media till the exponential phase and diluted to 0.1 OD (600 nm) in media. Different concentrations of fluconazole dissolved in media were exposed to the fungal suspension. The absorbance of the plate was recorded for 24 hours, with a reading every hour. The plate was maintained at 37 °C and protected from light during the experiment.

Growth Curve of SNAP Against *C. albicans*

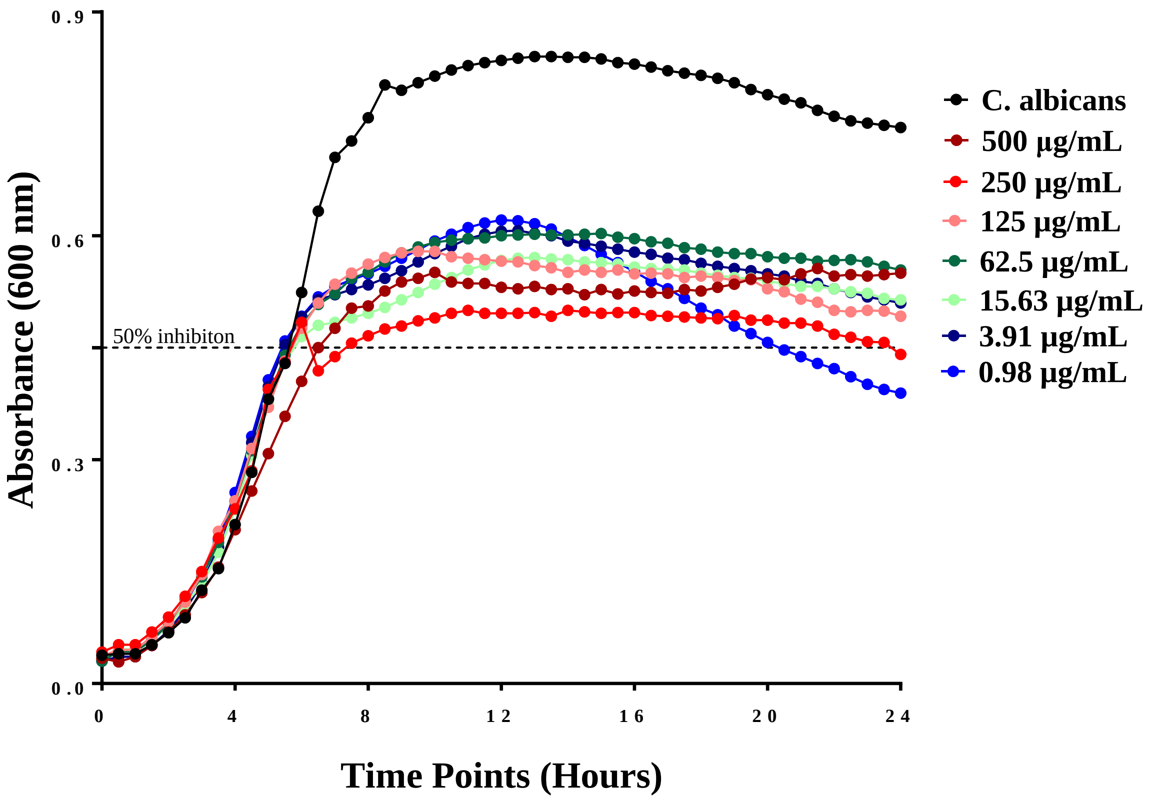

Figure S8: Growth curve of *C. albicans* treated with SNAP. *C. albicans* was cultured in yeast media till the exponential phase and diluted to 0.1 OD (600 nm) in media. Different concentrations of fluconazole dissolved in media were exposed to the fungal suspension. The absorbance of the plate was recorded for 24 hours, with a reading every hour. The plate was maintained at 37 °C and protected from light during the experiment.

Supporting Tables

Water Uptake of Fluconazole Incorporated Composites

Table S1: Percent Water Uptake of composites containing fluconazole and SNAP.

Sample	Percent Water Uptake
Fluconazole (1 *wt%*)	3.6 ± 0.4
Fluconazole (5 *wt%*)	3.7 ± 2.7
Fluconazole (10 *wt%*)	4.8 ± 2.2
Fluconazole (20 *wt%*)	4.1 ± 1.6
SNAP (10 *wt%*) + Fluconazole (1 *wt%*)	7.5 ± 4.4
SNAP (10 *wt%*) + Fluconazole (5 *wt%*)	6.6 ± 2.4
SNAP (10 *wt%*) + Fluconazole (10 *wt%*)	8.9 ± 2.1
SNAP (10 *wt%*) + Fluconazole (20 *wt%*)	5.3 ± 1.6

SNAP blended into TSPCU, fluconazole blended into TPU.

X ray Diffraction of Fluconazole Incorporated Composites

Table S2: XRD of composites containing fluconazole.

Sample	Total area of crystalline peaks	Total area	Crystallinity Index
TPU (SG80A)	6.93	13.28	52.23
Fluconazole (1 *wt%*)	7.00	12.86	54.44
Fluconazole (5 *wt%*)	8.17	13.34	61.21
Fluconazole (10 *wt%*)	7.80	12.17	65.72
Fluconazole (20 *wt%*)	8.52	12.50	66.02
Fluconazole (40 *wt%*)	7.19	10.31	69.71

SNAP blended into TSPCU, fluconazole blended into TPU.

Release of Fluconazole and SNAP from the polymeric matrix over 72 hours

Table S3: Daily release of SNAP and fluconazole from SNAP-fluconazole composites over 3 days under physiological conditions (37 °C, pH 7.4, submerged in PBS-EDTA)

Time Point	Fluconazole *1 wt%* (µg mL^{-1})	Fluconazole *5 wt%* (µg mL^{-1})	Fluconazole *10 wt%* (µg mL^{-1})	Fluconazole *20 wt%* (µg mL^{-1})	SNAP (µg) / Polymer (mg)
**24 hours**	12.0 ± 2.9	27.4 ± 3.3	28.5 ± 6.6	41.6 ± 0.3	7.5 ± 3.1
**48 hours**	7.7 ± 1.8	10.9 ± 2.3	14.9 ± 5.2	38.5 ± 5.4	5.4 ± 2.0
**72 hours**	4.1 ± 1.2	6.9 ± 5.6	9.2 ± 2.6	24.9 ± 3.1	3.9 ± 1.7

Antifungal activity of SNAP-Fluconazole polymeric composites

Table S4: Fungal reduction of NO and fluconazole-releasing samples against *C. albicans.*

**Sample**	**Change compared to control**	***C. albicans***	
		**Adhered**	**Planktonic**
SNAP (10 *wt%*)	Log Reduction	0.48 ± 0.16	0.28 ± 0.08
	Percent Reduction	64.28 ± 12.42	46.27 ± 9.67
Fluconazole (1 *wt%*)	Log Reduction	0.33 ± 0.11	0.24 ± 0.06
	Percent Reduction	51.18 ± 12.55	41.79 ± 7.31
Fluconazole (5 *wt%*)	Log Reduction	0.99 ± 0.11	0.51 ± 0.13
	Percent Reduction	89.58 ± 2.27	67.61 ± 8.65
Fluconazole (10 *wt%*)	Log Reduction	0.85 ± 0.12	0.53 ± 0.06
	Percent Reduction	86.26 ± 3.64	70.29 ± 4.36
Fluconazole (20 *wt%*)	Log Reduction	1.70 ± 0.27	0.92 ± 0.03
	Percent Reduction	97.66 ± 1.13	87.91 ± 0.97
SNAP (10 *wt%*) + Fluconazole (1 *wt%*)	Log Reduction	0.36 ± 0.06	0.36 ± 0.09
	Percent Reduction	55.91 ± 6.77	55.22 ± 9.67
SNAP (10 *wt%*) + Fluconazole (5 *wt%*)	Log Reduction	1.81 ± 0.31	0.61 ± 0.07
	Percent Reduction	98.07 ± 1.04	75.08 ± 4.24
SNAP (10 *wt%*) + Fluconazole (10 *wt%*)	Log Reduction	2.12 ± 0.17	0.69 ± 0.06
	Percent Reduction	99.17 ± 0.39	79.40 ± 2.69
SNAP (10 *wt%*) + Fluconazole (20 *wt%*)	Log Reduction	1.91 ± 0.32	1.11 ± 0.05
	Percent Reduction	98.42 ± 1.02	92.09 ± 0.92

Antibacterial activity of SNAP-Fluconazole polymeric composites

Table S5: Bacterial reduction of NO and fluconazole-releasing samples against *S. aureus* and *E. coli.*

Sample	Change compared to control	*E. coli*		*S. aureus*	
		Adhered	Planktonic	Adhered	Planktonic
SNAP (10 *wt%*)	Log Reduction	2.51 ± 0.14	1.53 ± 0.13	3.37 ± 0.20	3.71 ± 0.09
	Percent Reduction	99.68 ± 0.11	96.96 ± 0.81	99.95 ± 0.02	99.98 ± 0.00
Fluconazole (5 *wt%*)	Log Reduction	0.42 ± 0.10	0.05 ± 0.05	0.60 ± 0.12	0.11 ± 0.08
	Percent Reduction	60.63 ± 8.57	10.87 ± 11.09	73.93 ± 6.56	20.39 ± 14.65
Fluconazole (10 *wt%*)	Log Reduction	0.25 ± 0.26	0.12 ± 0.12	0.39 ± 0.13	0.29 ± 0.51
	Percent Reduction	33.54 ± 32.55	21.74 ± 19.19	58.07 ± 13.15	18.74 ± 61.78
Fluconazole (20 *wt%*)	Log Reduction	-0.13 ± 0.24	0.13 ± 0.05	0.07 ± 0.16	0.26 ± 0.31
	Percent Reduction	59.18 ± 95.25	26.09 ± 8.13	8.39 ± 30.33	29.69 ± 48.46
SNAP (10 *wt%*) + Fluconazole (5 *wt%*)	Log Reduction	2.14 ± 0.49	1.91 ± 0.21	3.16 ± 0.32	3.41 ± 0.09
	Percent Reduction	98.86 ± 0.82	98.62 ± 0.69	99.91 ± 0.05	99.96 ± 0.01
SNAP (10 *wt%*) + Fluconazole (10 *wt%*)	Log Reduction	1.87 ± 0.23	1.59 ± 0.22	3.61 ± 0.10	3.18 ± 0.04
	Percent Reduction	98.38 ± 1.15	97.11 ± 1.47	99.97 ± 0.01	99.93 ± 0.01
SNAP (10 *wt%*) + Fluconazole (20 *wt%*)	Log Reduction	2.02 ± 0.15	1.94 ± 0.15	4.02 ± 0.28	3.16 ± 0.11
	Percent Reduction	98.99 ± 0.31	98.79 ± 0.40	99.99 ± 0.01	99.93 ± 0.02

References

(1) Gu, X.; Jiang, W. Characterization of polymorphic forms of fluconazole using fourier transform Raman spectroscopy. *Journal of pharmaceutical sciences* **1995**, *84* (12), 1438-1441.
